# Real-time monitoring of subcellular H_2_O_2_ distribution in *Chlamydomonas reinhardtii*

**DOI:** 10.1093/plcell/koab176

**Published:** 2021-07-01

**Authors:** Justus Niemeyer, David Scheuring, Julian Oestreicher, Bruce Morgan, Michael Schroda

**Affiliations:** 1 Molekulare Biotechnologie & Systembiologie, TU Kaiserslautern, Paul-Ehrlich Straße 23, D-67663 Kaiserslautern, Germany; 2 Phytopathologie, TU Kaiserslautern, Paul-Ehrlich Straße 22, D-67663 Kaiserslautern, Germany; 3 Institute of Biochemistry, Zentrum für Human und Molekularbiologie (ZHMB), Saarland University, D-66123 Saarbrücken, Germany

## Abstract

Hydrogen peroxide (H_2_O_2_) is recognized as an important signaling molecule in plants. We sought to establish a genetically encoded, fluorescent H_2_O_2_ sensor that allows H_2_O_2_ monitoring in all major subcompartments of a *Chlamydomonas* cell. To this end, we used the *Chlamydomonas* Modular Cloning toolbox to target the hypersensitive H_2_O_2_ sensor reduction–oxidation sensitive green fluorescent protein2-Tsa2ΔC_R_ to the cytosol, nucleus, mitochondrial matrix, chloroplast stroma, thylakoid lumen, and endoplasmic reticulum (ER). The sensor was functional in all compartments, except for the ER where it was fully oxidized. Employing our novel sensors, we show that H_2_O_2_ produced by photosynthetic linear electron transport (PET) in the stroma leaks into the cytosol but only reaches other subcellular compartments if produced under nonphysiological conditions. Furthermore, in heat-stressed cells, we show that cytosolic H_2_O_2_ levels closely mirror temperature up- and downshifts and are independent from PET. Heat stress led to similar up- and downshifts of H_2_O_2_ levels in the nucleus and, more mildly, in mitochondria but not in the chloroplast. Our results thus suggest the establishment of steep intracellular H_2_O_2_ gradients under normal physiological conditions with limited diffusion into other compartments. We anticipate that these sensors will greatly facilitate future investigations of H_2_O_2_ biology in plant cells.

## Teaser

The establishment of a hypersensitive H_2_O_2_ sensor in six major compartments of the *Chlamydomonas* cell reveals steep intracellular H_2_O_2_ gradients under normal physiological conditions with limited diffusion into other compartments.

##  

**Figure:**
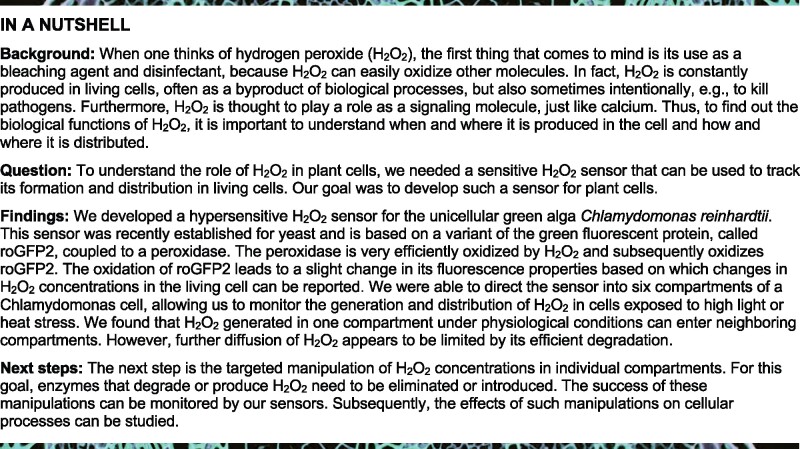


## Introduction

In plant cells, hydrogen peroxide (H_2_O_2_), or its precursor superoxide ($O_{2}^{•–}$), is produced as a side-product of cellular processes including photosynthetic linear electron transport (PET), the mitochondrial respiratory chain, or substrate oxidation, for example by the photorespiration enzyme glycolate oxidase (Cheeseman, 2007; Foyer and Noctor, 2016). H_2_O_2_ is a relatively stable molecule and is largely unreactive with most proteins. It is therefore well-suited as a second messenger in cellular signaling cascades (Waszczak et al., 2018). Such signaling cascades can be fueled either with H_2_O_2_ produced as a side-product, e.g. from PET (Exposito-Rodriguez et al., 2017), or with H_2_O_2_ produced specifically for a signaling cascade. One such example is the respiratory burst oxidase homolog D (RBOHD) located in the plasma membrane that produces H_2_O_2_ for local and systemic signaling upon stimulation by wounding, cold, heat, high light, and salinity (Miller et al., 2009; Lew et al., 2020). RBOHD also plays a role during abscisic acid-mediated stomatal movement (Pei et al., 2000; Kwak et al., 2003).

The recognition of H_2_O_2_ as a second messenger has generated a demand for tools to measure H_2_O_2_ dynamics within plant cells. While the development of synthetic dyes for H_2_O_2_ detection has proven useful, several problems are inherently associated with their use, including variable uptake and efflux, lack of subcellular compartment specificity, lack of redox species specificity, and the irreversibility of their reaction with H_2_O_2_ (Bilan and Belousov, 2018; Roma et al., 2018). A major methodological advance for the subcellular detection of H_2_O_2_ came with the creation of genetically encoded fluorescent protein sensors that allow for subcellular compartment-specific monitoring of H_2_O_2_ in real time in living organisms. The first of these sensors was HyPer (Belousov et al., 2006). HyPer (from Hydrogen Peroxide) consists of a circularly permuted (cp) yellow fluorescent protein (YFP) positioned between the two halves of the H_2_O_2_-sensitive *Escherichia* *coli* transcription factor OxyR. The presence of H_2_O_2_ leads to the formation of a disulfide bond between the H_2_O_2_-reactive cysteine on one OxyR domain and the resolving cysteine residue on the other domain. This disulfide bond results in a structural change that is transmitted to cpYFP, thereby inducing a ratiometric change in its fluorescence excitation spectrum and allowing measurements that are independent of probe concentration. Several improved versions of HyPer have been generated since that expand its dynamic range, its oxidation and reduction rates, or employ a red fluorescent protein (Bilan and Belousov, 2018).

A second family of genetically encoded fluorescent H_2_O_2_ sensors is based on reduction–oxidation sensitive green fluorescent protein (roGFP2), which contains two cysteines in adjacent β-strands on the surface of the protein β-barrel (Hanson et al., 2004). The formation of a disulfide bond between these cysteines results in small structural changes that induce a ratiometric change in the fluorescence excitation spectrum. As with HyPer, roGFP2 allows measurements that are independent of sensor concentration. Since the cysteines of roGFP2 do not readily react with H_2_O_2_, roGFP2 needs to be coupled with an H_2_O_2_-reactive enzyme in a redox relay system. The first such sensor was roGFP2-Orp1, which involves a fusion between roGFP2 and the glutathione peroxidase-like enzyme Orp1 from budding yeast (*Saccharomyces cerevisiae*), with a short interspacing polypeptide linker (Gutscher et al., 2009). In this sensor, Orp1 sensitively reacts with H_2_O_2_ to form an intramolecular disulfide bond between its peroxidatic and resolving cysteine, followed by a thiol-disulfide exchange reaction to roGFP2.

Many cellular peroxiredoxins contain peroxidatic cysteine residues that react with H_2_O_2_ two to three orders of magnitude faster than that of the Orp1 peroxidase (Roma et al., 2018). This observation led to the generation of roGFP2-Tsa2ΔC_R_, a fusion between the typical yeast 2-Cys peroxiredoxin Thiol-Specific Antioxidant 2 (Tsa2) and roGFP2 (Morgan et al., 2016). The resolving cysteine in Tsa2 was mutated to alanine such that the reduction of the sensor by thioredoxin is impeded and sensor sensitivity further enhanced. In yeast, this sensor was shown to be approximately 20-fold more sensitive towards H_2_O_2_ than either HyPer or roGFP2-Orp1 (Morgan et al., 2016). Furthermore, roGFP2-Tsa2ΔC_R_ makes a negligible contribution to the cellular H_2_O_2_-scavenging capacity, has a low propensity for hyperoxidation, and is unaffected by changes in pH between 6.0 and 8.5 (Morgan et al., 2016; Roma et al., 2018). Its pH insensitivity distinguishes roGFP2-Tsa2ΔC_R_ from HyPer, for which additional pH probes need to be implemented to account for changes in pH, as observed for example in the stroma upon illumination (Schwarzlander et al., 2014; Exposito-Rodriguez et al., 2017). A pH-insensitive version of HyPer (HyPer7) has recently been established, but has not been tested in plant cells (Pak et al., 2020).

The aim of this work was to establish a sensitive H_2_O_2_ sensor for the unicellular green alga *Chlamydomonas* (*Chlamydomonas reinhardtii*), a traditional model system for plant cell biology and photosynthesis (Sasso et al., 2018). To this end, we engineered roGFP2-Tsa2ΔC_R_ as a genetic part for the *Chlamydomonas* Modular Cloning (MoClo) toolbox for synthetic biology. We targeted the sensor to six cell compartments: the cytosol, nucleus, mitochondrial matrix, chloroplast stroma, thylakoid lumen, and endoplasmic reticulum (ER); and show that changes in H_2_O_2_ concentrations can be monitored in real time in all compartments, except for the ER. Our data also revealed the establishment of strong intracellular H_2_O_2_ gradients in response to physiological stresses.

## Results

### Construction of an H_2_O_2_ sensor for *Chlamydomonas*

**Figure 1:**
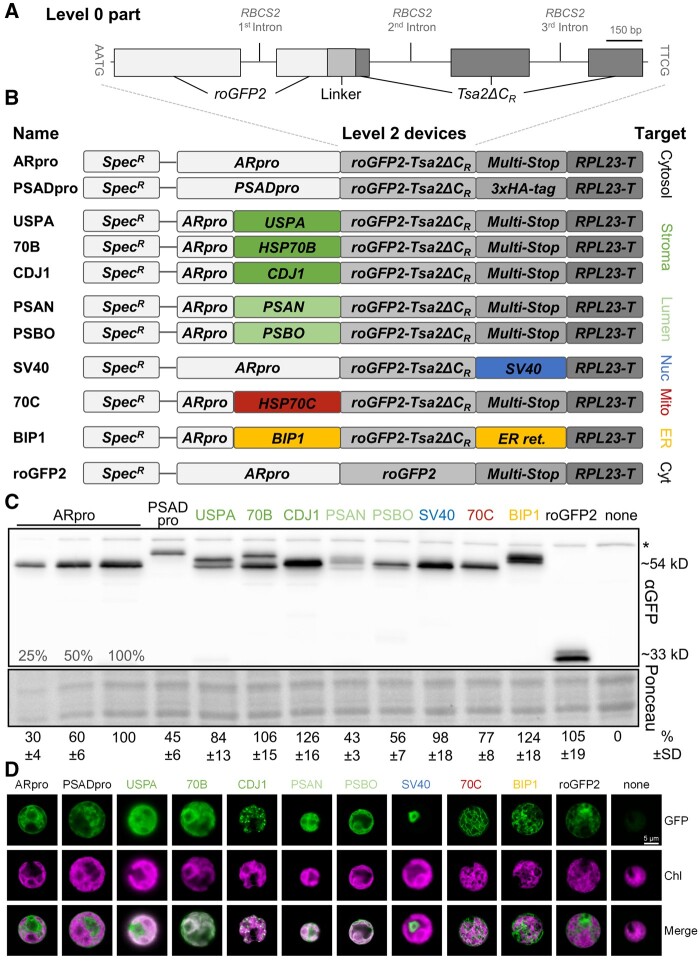
Targeting roGFP2 sensors to various compartments of a *Chlamydomonas* cell. A, Schematic representation of the level 0 part of the roGFP2-Tsa2ΔC_R_ H_2_O_2_ sensor. The 2,132-bp coding region (exons shown as boxes), interrupted by the three *RBCS2* introns (thin lines), was synthesized with optimal *Chlamydomonas* codon usage. RoGFP2 (light gray) is separated from Tsa2ΔC*_R_* (dark gray) by a linker (gray). B, Using MoClo, the *roGFP2-Tsa2ΔC_R_* part or only the *roGFP2* part was equipped with the *HSP70A-RBCS2* promotor (*ARpro*) or the *PSAD* promoter (*PSADpro*) and the *RPL23* terminator (*RPL23-T*) as well as various sequences adding N- or C-terminal targeting signals to target the sensors to the cytosol (cyt), the stroma, the thylakoid lumen (lumen), the nucleus (nuc), mitochondria (mito), or the ER. The resulting level 1 modules were combined with another level 1 module comprising the *aadA* resistance marker flanked by the *PSAD* promoter and terminator (*Spec^R^*) to yield the 11 level 2 devices shown. C, Comparison of roGFP2 accumulation levels in transformants generated with the level 2 devices shown in (B). The transformants shown represent those with highest protein accumulation levels among at least 13 independent transformants screened for each construct. Total cell protein extracts corresponding to 1.5-µg chlorophyll for each transformant and the UVM4 recipient strain (none) were separated by SDS–PAGE and analyzed by immunoblotting using an antibody against GFP. Signals retrieved from three independent experiments were quantified and normalized to the signal obtained with the transformant harboring a construct driving the expression of *roGFP2-Tsa2ΔC_R_* (encoding a cytosolic sensor) from the *AR promoter*, which was set to 100%. Mean values are given below the panel (± sd, *n*=3). A representative experiment is shown, with Ponceau staining demonstrating equal loading. The asterisk indicates a nonspecific cross-reaction with the GFP antibody. D, Representative confocal microscopy images of individual cells of the untransformed UVM4 strain (none) and the transformants analyzed in (C). Shown are GFP fluorescence, chlorophyll autofluorescence (Chl) and both signals merged. About 4–50 individual cells were analyzed per transformant strain, with consistent localization (Supplemental Table S3).

To develop a genetically encoded, fluorescent H_2_O_2_ sensor for *Chlamydomonas*, we synthesized the coding sequence of *roGFP2-Tsa2ΔC_R_* (Morgan et al., 2016) with codons optimized for expression in *Chlamydomonas* and containing the three introns of the nuclear RuBisCO small subunit gene *RBCS2*, as a standard gene part (level 0) for the *Chlamydomonas* MoClo kit (Crozet et al., 2018; Figure 1A). The MoClo strategy is based on Type IIS restriction enzymes and allows the directed assembly of standard gene parts (promoters, coding sequences, untranslated regions) in a single reaction into modules (transcriptional units, level 1; Weber et al., 2011). Likewise, level 1 modules can be further assembled into multigene constructs termed devices (level 2). Following this strategy, the *roGFP2-Tsa2ΔC_R_* part was assembled into level 1 modules. These included either the *PHOTOSYSTEM I SUBUNIT D* (*PSAD*) promoter (*PSADpro*) or the *HEAT SHOCK PROTEIN 70A* (*HSP70A*)*-RBCS2* (*AR*) fusion promoter (*ARpro*), the *RIBOSOMAL PROTEIN L23* (*RPL23*) terminator, various sequences encoding N-terminal targeting peptides or retention signals to facilitate localization to defined subcellular compartments, and in one case the sequence of a C-terminal 3xHA tag. The resulting level 1 modules were then assembled into level 2 devices together with a spectinomycin resistance cassette (*aadA*) under the control of the *PSAD* promoter and terminator (Figure 1B). We then transformed the level 2 devices into the UVM4 expression strain (Neupert et al., 2009). As a control, we generated a level 0 part containing only the *roGFP2* coding sequence, harboring the first *RBCS2* intron, and placed it under control of the *AR* promoter (roGFP2, Figure 1B). The best promoters and targeting peptides to use in *Chlamydomonas* are still a subject of debate. We therefore tested different promoters and targeting peptide combinations. For each device, we picked at least 13 spectinomycin-resistant transformants and screened them for sensor accumulation by immunoblotting with an anti-green fluorescent protein (GFP) antibody. Between 23% and 70% of the transformants accumulated the sensor protein to readily detectable levels and with the expected size (Supplemental Figure S1). To compare the accumulation levels of the various sensors, we separated whole-cell protein extracts from the best accumulating transformants on the same sodium dodecyl sulphate–polyacrylamide gel electrophoresis (SDS–PAGE) and probed the resulting immunoblot using an anti-GFP antibody (Figure 1C).

With respect to the constructs for cytosolic localization, we observed that the sensor driven by the *AR* promoter accumulated to approximately two-fold higher levels than the *PSAD* promoter-driven sensor (Figure 1C; the size shift seen for the *PSAD* promoter-driven sensor comes from the 3xHA tag). Furthermore, unfused roGFP2 accumulated to about the same level as the full roGFP2-Tsa2ΔC_R_ sensor (Figure 1C). For targeting to the mitochondrial matrix, ER, and nucleus, we employed targeting sequences from HSP70C, binding immunoglobulin protein (BiP1), and the nuclear localization signal from Simian Virus 40 (SV40), respectively. For each construct, we detected a single protein band by immunoblotting and confirmed the correct localization of the sensor by confocal fluorescence microscopy (Figure 1, C and D). For targeting to the chloroplast stroma, we tested three different transit peptides derived from universal stress protein A (USPA), HSP70B, and chloroplast DnaJ homolog 1 (CDJ1). We obtained a double band for all USPA transformants and detected some GFP fluorescence in the cytosol (Figure 1, C and D; Supplemental Figure S1). We therefore reasoned that the USPA transit peptide was not efficiently driving chloroplast import. Likewise, as judged from the protein double band and residual GFP fluorescence in the cytosol, the HSP70B transit peptide was also unable to efficiently drive sensor import into chloroplasts (Figure 1, C and D). In contrast, we observed a single protein band in immunoblots and an exclusive chloroplast localization, as judged by fluorescence microscopy, when using the CDJ1 transit peptide (Figure 1, C and D). We observed a punctate signal for the stromal sensor that might be derived from heterooligomerization with (a) native peroxidase(s), as was reported in yeast (Morgan et al., 2016). We noticed no punctate signal in a transformant with weaker sensor accumulation (Supplemental Figure S2, A and B). We also wished to target the sensor to the thylakoid lumen; to this end, we created level 0 parts encoding the bipartite signaling sequences from the PHOTOSYSTEM I SUBUNIT N (PSAN) and PHOTOSYSTEM II SUBUNIT O (PSBO) proteins. PSAN uses the twin arginine protein translocation (Tat) pathway for the import of folded proteins, while PSBO uses the Sec pathway for the import of unfolded proteins (Albiniak et al., 2012). Immunological detection of the sensor in the highest accumulating *PSAN* and *PSBO* transformants showed that the sensor reaches only about half of the levels detected with the best *ARpro* transformant. Moreover, we obtained multiple protein bands that migrated with larger apparent mass than expected for the mature protein in *PSAN* transformants (Figure 1C;Supplemental Figure S1). We also observed GFP fluorescence all over the chloroplast and even in the cytosol (Figure 1D), indicating incomplete import of the sensor into both chloroplast and thylakoid lumen by the PSAN bipartite targeting sequence. In contrast, we observed only one major protein band migrating with the expected mass in *PSBO* transformants, with GFP fluorescence restricted to the thylakoid lumen, indicating full functionality of the PSBO bipartite targeting signal (Figure 1, C and D;Supplemental Figure S1).

### RoGFP2-Tsa2ΔC_R_ functions as an ultra-sensitive H_2_O_2_ sensor in *Chlamydomonas*

**Figure 2:**
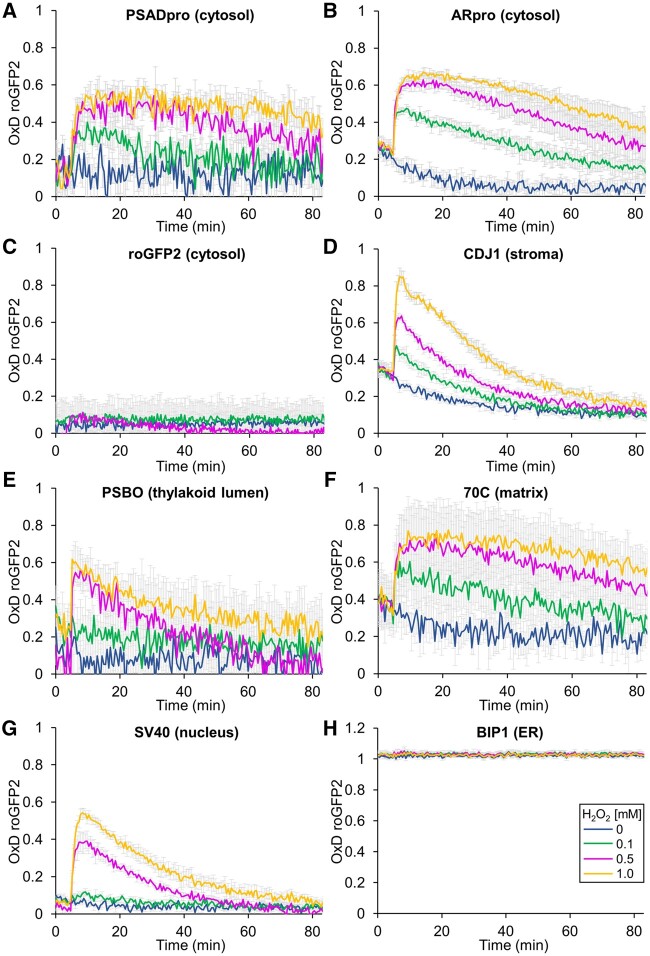
Real-time monitoring of H_2_O_2_ levels in different subcellular compartments under steady-state conditions and after the addition of H_2_O_2._ A–H, Fluorescence measurement of roGFP2-Tsa2ΔC_R_ and roGFP2 in transformant strains shown in Figure 1, C and D under steady-state conditions (no H_2_O_2_ added, blue) and after the addition of H_2_O_2_ at concentrations of 0.1 mM (green), 0.5 mM (magenta), and 1 mM (yellow). Values were calculated relative to those obtained for fully reduced (0) and fully oxidized (1) sensors. Shown are means of three independent experiments, error bars represent standard deviation. Sensors were targeted to the cytosol (A–C), the chloroplast stroma (D), the chloroplast lumen (E), the mitochondrial matrix (F), the nucleus (G), or the ER (H).

Having generated a suite of transformants targeting the sensor to distinct subcellular compartments, we first tested whether we could detect the cytosolic roGFP2-Tsa2ΔC_R_ sensor and monitor its oxidation by exogenously applying H_2_O_2_ in real time. To this end, we took cells of the highest accumulating *PSADpro* transformant. We concentrated cells by centrifugation in a 96-well microtiter plate and monitored GFP fluorescence in control samples (with no added H_2_O_2_) and upon the addition of 0.1-, 0.5-, or 1-mM H_2_O_2_ in a fluorescence plate reader. Note that these H_2_O_2_ concentrations are initial exogenous concentrations; in HeLa cells, the resulting intracellular H_2_O_2_ concentrations were estimated to be several 100-fold lower (Huang and Sikes, 2014). As shown in Figure 2A, we indeed detected sensor oxidation in a dose-dependent manner, but the signal was very noisy. This noise was mainly derived from the fluorescence signal gained from excitation at 405 nm, where the difference between oxidized and reduced sensor is considerably smaller than at 488 nm (Meyer and Dick, 2010; Supplemental Figure S3). However, we observed a clear H_2_O_2_ concentration-dependent sensor response in the *ARpro* transformant (Figure 2B), presumably because the sensor accumulated to about two-fold higher levels than in the *PSADpro* transformant. The cytosolic roGFP2-Tsa2ΔC_R_ sensor was ∼25% oxidized at steady state at the beginning of our assays. Upon H_2_O_2_ addition, we observed a rapid probe oxidation, which slowly recovered over a period of ∼80 min, presumably in a glutathione (GSH)/glutaredoxin-dependent manner, as previously shown in yeast (Morgan et al., 2016). The unfused cytosolic roGFP2 was completely unresponsive to the same concentrations of H_2_O_2_ used above, confirming the requirement of the Tsa2 moiety to couple roGFP2 oxidation with changes in H_2_O_2_ availability (Figure 2C). Notably, the kinetics of roGFP2-Tsa2ΔC_R_ oxidation and reduction were very similar to those observed in yeast (Morgan et al., 2016), including a reduction of the sensor over time in untreated samples, which in yeast was shown to be caused by cell-dependent oxygen consumption from the assay buffer. That this is also true for *Chlamydomonas* is suggested by the observation that the reduction of the sensor occurs faster at higher cell numbers used in the assay, just as in yeast (Supplemental Figure S4). These data strongly suggest that roGFP2-Tsa2ΔC_R_ functions as an ultra-sensitive sensor in *Chlamydomonas* and is capable of sensing and responding to changes in basal cytosolic H_2_O_2_ levels, as it does in yeast.

For comparison, we synthesized the coding sequence of the roGFP2-Orp1 sensor (Gutscher et al., 2009) with the *Chlamydomonas* codon usage and with three *RBCS2* introns as a level 0 construct, equipped it with the *AR* promoter and *RPL23* terminator in a level 1 construct, and at level 2 with the *aadA* cassette (Supplemental Figure S5, A and B). Transformants accumulated the roGFP2-Orp1 sensor in the cytosol up to ∼86% of levels of roGFP2-Tsa2ΔC_R_ in the highest accumulating *ARpro* transformant (Supplemental Figure S5, C and D). The sensor was fully reduced under steady-state conditions and was barely oxidized when H_2_O_2_ was added exogenously. These results clearly demonstrate the superior sensitivity of the roGFP2-Tsa2ΔC_R_ sensor compared to the roGFP2-Orp1 sensor in *Chlamydomonas* (Supplemental Figure S5, E and F).

Having confirmed the feasibility of H_2_O_2_ measurements with cytosolic roGFP2-Tsa2ΔC_R_, we next tested the response to exogenously added H_2_O_2_ of the sensor targeted to the five additional cellular compartments. Under steady-state conditions, stromal and mitochondrial sensors were more oxidized than those in the cytosol or nucleus (Figure 2, B, D, F, and G). However, as for the cytosolic sensor, we observed a rapid dose-dependent oxidation followed by slow reduction in response to exogenous H_2_O_2_ for sensors targeted to the chloroplast stroma, thylakoid lumen, mitochondrial matrix, and the nucleus (Figure 2, D–G). The response for the stromal sensor accumulating to lower levels and not producing fluorescent puncta was very similar to that of the highly accumulating sensor, although much more noisy (Supplemental Figure S2C). Measurements were not possible in the ER, as the sensor was fully oxidized at steady state (Figure 2H). In general, signal noise inversely correlated with the accumulation level of the sensor: the highest noise was detected for the thylakoid lumen sensor, whose protein levels were about two-fold less abundant than those for the sensors in the stroma and nucleus, which showed the least noise (Figure 1, C, D, E, and G). The nuclear, stromal, and thylakoid lumen-localized sensors were reduced at faster rates than their counterparts in the cytosol and mitochondrial matrix (Figure 2, B, D–G). Overall, the cytosolic sensor exhibited the highest sensitivity to exogenously added H_2_O_2_ and the nuclear sensor the lowest, as judged from their responsiveness to the lowest H_2_O_2_ concentration employed (0.1 mM; Figure 2, B and G). This result may indicate that H_2_O_2_ scavenging enzymes in the cytosol limit the diffusion of exogenous H_2_O_2_ to the intracellular organelles.

### RoGFP2-Tsa2ΔC_R_ reveals the formation of light-dependent subcellular H_2_O_2_ gradients

**Figure 3:**
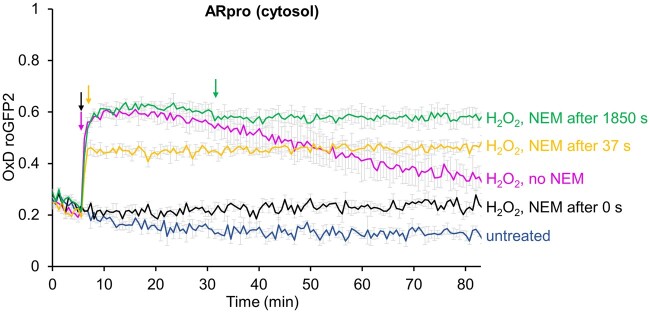
Verification of efficient trapping of the sensor oxidation state with NEM. *Chlamydomonas* cells accumulating roGFP2-Tsa2ΔC_R_ in the cytosol were treated with 0.5-mM H_2_O_2_ (magenta) and 16.67-mM NEM was added with time differences of 0 s (black), 37 s (yellow), and 1850 s (green). No H_2_O_2_ was added in the control (blue). Fluorescence was recorded and values were calculated relative to those obtained for fully reduced (0) and fully oxidized (1) sensors. Shown are means of three independent experiments, error bars represent standard deviation.

**Figure 4:**
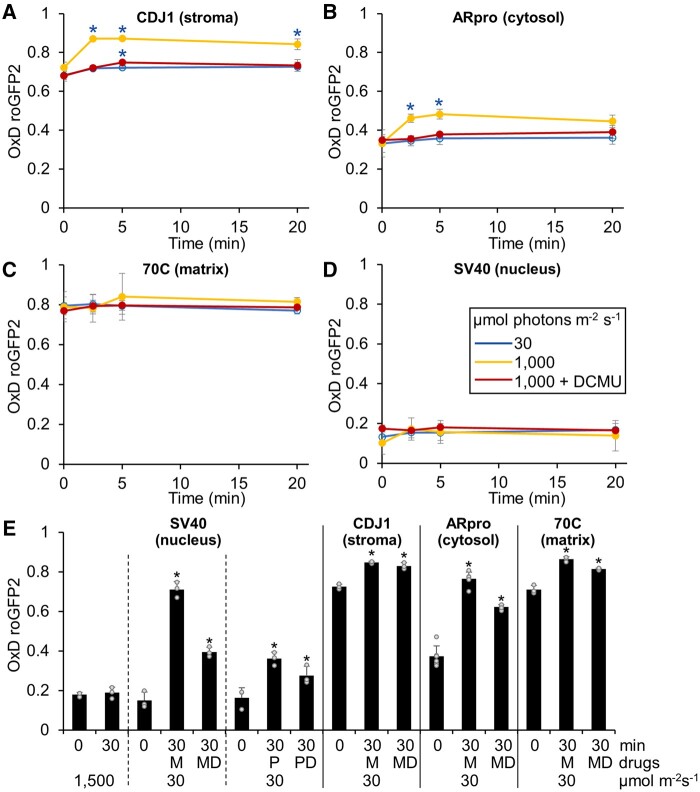
Monitoring H_2_O_2_ levels in different subcellular compartments under high-light exposure and after the application of metronidazole or paraquat. *Chlamydomonas* transformant cells, accumulating roGFP2-Tsa2ΔC_R_ in the stroma (A), cytosol (B), mitochondrial matrix (C), or nucleus (D) were kept in a low light intensity of 30 µmol photons m^−2^ s^−1^ (blue) or exposed to high light of 1,000 µmol photons m^−2^ s^−1^ in the absence (yellow) or presence (red) of 10 µM DCMU. The oxidation state of the sensor was trapped by the addition of NEM and roGFP2 fluorescence was measured in a plate reader. Shown are mean values from three independent experiments, error bars represent standard deviation. Asterisks indicate significant differences with respect to the low light control (two-tailed, unpaired *t* test with Bonferroni–Holm correction, *P* < 0.05). The absence of an asterisk means that there were no significant differences. E, Transformant cells accumulating roGFP2-Tsa2ΔC_R_ in the indicated compartments were grown in low light of 30 µmol photons m^−2^ s^−1^ (0) and then exposed to 1,500 µmol photons m^−2^ s^−1^ for 30 min, or kept at low light for 30 min in the presence of 2-mM metronidazole (M) or 1-µM paraquat (P), alone or together with 10-µM DCMU (MD, PD). The oxidation state of the sensor was trapped by the addition of NEM and roGFP2 fluorescence was measured in a plate reader. Error bars represent standard deviation from three independent experiments. Asterisks indicate significant differences with respect to the low light control (two-tailed, unpaired *t* test with Bonferroni–Holm correction, *P* < 0.05). The absence of an asterisk means that there were no significant differences.

With these *Chlamydomonas* lines with sensors in different subcellular compartments, we asked whether we could detect changes in H_2_O_2_ levels under challenging environmental conditions. We intended to first test whether exposing cells to high light would result in increased H_2_O_2_ levels in the chloroplast stroma and whether H_2_O_2_ would diffuse into other subcellular compartments. The fluorescence assay used up to this point allowed for monitoring of the sensor oxidation state in real time. However, as we had no way to illuminate cells in our plate reader assay, we turned to a “redox trapping” approach. We adopted a protocol from yeast that allows for a rapid “trapping” of the sensor oxidation state with the membrane permeable alkylating agent N-ethylmaleimide (NEM; Morgan et al., 2016). NEM irreversibly alkylates free thiol groups, thus preventing further probe oxidation or reduction. To test the applicability of NEM trapping for *Chlamydomonas*, we added H_2_O_2_ exogenously to the *ARpro* transformant, followed by the addition of NEM immediately (0 s), after 37 s and after 1,850 s, and monitored sensor oxidation over time. As shown in Figure 3, the oxidation state of the sensor was instantaneously trapped after NEM addition and remained stable over the time course of the experiment, thus demonstrating the applicability of the NEM trapping in *Chlamydomonas*. With this method at hand, we next grew transformants accumulating the sensor in the cytosol, nucleus, matrix, stroma, and thylakoid lumen under a low light intensity of 30 µmol photons m^−2^ s^−1^ to mid-log phase. Cultures were then kept in low light or exposed to 1,000 µmol photons m^−2^ s^−1^ in the absence or the presence of 3-(3,4-dichlorophenyl)-1,1-dimethylurea (DCMU) to block PET. We harvested cells before and during the time course and measured the oxidation state of the sensor by plate reader after the NEM trapping. In these experiments (Figure 4), we observed a higher oxidation state of the sensor under steady-state conditions in all compartments when compared to the experiments shown in Figure 2, for which oxygen consumption of cells during centrifugation and preparation for the plate reader is likely to result in lower cellular H_2_O_2_ levels. Hence, the NEM trapping of cells directly from the culture circumvented this technical limitation and revealed the true steady-state probe oxidation. Upon shifting cells into high light, H_2_O_2_ levels in the stroma increased rapidly within minutes and remained at higher levels when compared to the cells kept under low light intensity during the 20-min time course (Figure 4A). The cytosolic probe also responded rapidly, with a nonsignificant trend for probe recovery after 20 min. With slower kinetics, we also observed a slight, nonsignificant trend towards higher H_2_O_2_ levels in high light in the mitochondrial matrix, while we failed to detect an increase in the nucleus (Figure 4, C and D). The increase in H_2_O_2_ levels in all tested compartments was largely abolished when cells were exposed to high light in the presence of DCMU, indicating that H_2_O_2_ in high light was produced by PET. Because of its low signal-to-noise ratio (Figure 2E), we were unable to monitor the response of the thylakoid lumen sensor in this assay.

That we did not detect any increase in H_2_O_2_ levels in the nucleus of *Chlamydomonas* cells exposed to high light appeared surprising because such an increase was previously observed in *Nicotiana benthamiana* epidermal cells exposed to 1,000 µmol photons m^−2^ s^−1^ (Exposito-Rodriguez et al., 2017). We therefore repeated our experiment at higher light intensities of 1,500 µmol photons m^−2^ s^−1^ for 30 min but again were unable to detect any increase of H_2_O_2_ levels in the nucleus (Figure 4E).

Metronidazole and paraquat (also known as methyl viologen) both facilitate the Mehler reaction in intact cells, i.e., the transfer of electrons from PSI to oxygen to produce superoxide, which is then converted to H_2_O_2_ by superoxide dismutase (Mehler, 1951; Asada, 2000). While paraquat is directly reduced by PSI, metronidazole is reduced by ferredoxin (Schmidt et al., 1977). Paraquat is active at much lower concentrations than metronidazole. However, in contrast to metronidazole, paraquat can kill *Chlamydomonas* cells even when grown in the dark and therefore must affect other cellular processes in addition to PET (Schmidt et al., 1977). We employed both drugs to test whether the enhanced rates of H_2_O_2_ produced in their presence even under low light intensities might be detected in the nucleus. As shown in Figure 4E, this was indeed the case following 30-min incubation with either drug. We will note that such short treatment leads to a growth retardation but does not kill the cells (Supplemental Figure S6). We also monitored H_2_O_2_ production upon metronidazole treatment in the stroma, cytosol, and matrix and discovered that H_2_O_2_ levels increase in all subcompartments. In all cases, DCMU decreased the accumulation of H_2_O_2_ but did not abolish it, most likely because plastoquinone reduction occurs from starch breakdown via NADH dehydrogenase 2 (Ndh2) mediating the so-called pseudo-linear electron transport under these conditions (Bulté et al., 1990; Mus et al., 2005; Desplats et al., 2009). Similar lower H_2_O_2_ levels were also observed in Arabidopsis seedlings treated with paraquat and DCMU in the light (Ugalde et al., 2021).

In summary, our data show a rapid, PET-dependent increase in H_2_O_2_ levels in the chloroplast stroma. While H_2_O_2_ levels produced by metronidazole or paraquat feeding were so high that H_2_O_2_ readily diffused into all other subcompartments, H_2_O_2_ production under high light was of sufficient magnitude to increase cytosolic H_2_O_2_ but did not substantially influence H_2_O_2_ levels in the mitochondrial matrix or nucleus. Thus, these results are consistent with our exogenous H_2_O_2_ application experiments, suggesting that the cytosol acts as an effective barrier that limits the intracellular diffusion of H_2_O_2_ concentrations typically produced under physiological conditions.

### Heat stress-derived H_2_O_2_ is independent of photosynthetic electron transport

**Figure 5:**
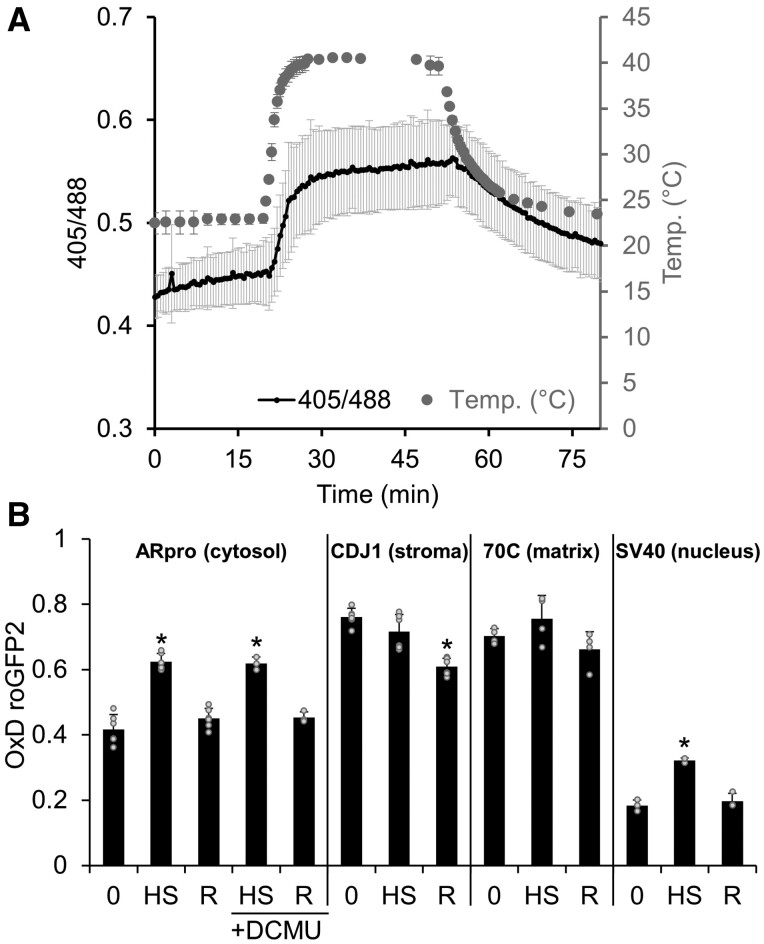
Monitoring H_2_O_2_ levels during heat stress and recovery. A, *Chlamydomonas* cells accumulating roGFP2-Tsa2ΔC_R_ in the cytosol were grown in a low light intensity of 30 µmol photons m^−2^ s^−1^ for 20 min, exposed to 40°C for 30 min, and shifted back to 23°C for 30 min. The ratio of the fluorescence light emitted after excitation at 405 and 488 nm, respectively, was monitored in real time using a spectrofluorometer (black line). The temperature in the culture was monitored in parallel (gray circles). Shown are mean values from three independent experiments, error bars represent standard deviation. B, Transformant cells accumulating roGFP2-Tsa2ΔC_R_ in the cytosol, stroma, matrix, or nucleus were grown in a low light intensity of 30 µmol photons m^−2^ s^−1^ at 23°C (0), exposed to 40°C for 30 min (HS), and shifted back to 23°C for another 30 min (R). If indicated, cultures were supplied with 10-µM DCMU before the experiment was started. The oxidation state of the sensor was trapped by the addition of NEM and roGFP2 fluorescence was measured in a plate reader. Error bars represent standard deviation from three independent experiments. Asterisks indicate significant differences with respect to the 23°C (0) control (two-tailed, unpaired *t* test with Bonferroni–Holm correction, *P* < 0.01). The absence of an asterisk means that there were no significant differences (*P* ≥ 0.05).

We chose heat stress as a second environmental challenge. To test whether H_2_O_2_ levels within *Chlamydomonas* cells changed when exposed to heat stress, we employed the cell line accumulating the H_2_O_2_ sensor in the cytosol, as this sensor exhibited the highest signal-to-noise ratio (Figure 2B). To allow us to monitor real-time changes in cytosolic H_2_O_2_ during heat stress, we developed an experimental setup in which cells were cultivated under agitation and continuously circulated to a quartz cuvette inserted into a spectrofluorometer via connected tubing and a peristaltic pump. The culture flask, equipped with a temperature sensor, was kept on a platform well above the water surface of a 40°C water bath, placed into the water bath 20 min after the fluorescence recording had started, and placed back onto the platform 30 min later for recovery. Notably, sensor oxidation and thus H_2_O_2_ production occurred immediately after the temperature increased and immediately declined when the temperature dropped (Figure 5A). When the cultures reached the initial starting temperature 30 min into recovery, probe oxidation had not fully recovered. To verify that the change in fluorescent light emission under heat stress was not caused by a heat-induced conformational change of the sensor, we trapped the sensor with NEM in steady state as well as in the fully oxidized and fully reduced states and recorded fluorescent light emission after excitation at 405 nm and 488 nm at 25°C, 30°C, 35°C, 40°C, and 45°C. As shown in Supplemental Figure S7, we observed no change in fluorescence ratios, indicating that the sensor is stable at this range of temperatures and that the changes in fluorescence emission under heat stress are indeed caused by changes in roGFP2 oxidation.

As a further test of our observations, we used the same setup employed for the high light experiments (Figure 4). Specifically, we harvested cells from a culture accumulating the cytosolic sensor before shifting the temperature from 23°C to 40°C, after exposure to 40°C for 30 min, and 30 min after shifting the culture back to 23°C. Again, we measured the redox state of the sensor after trapping with NEM. As shown in Figure 5B, we confirmed that heat stress leads to a transient increase in sensor oxidation that was abolished when the temperature dropped again. To rule out that the heat-induced increase in sensor oxidation is due to an impairment of reduction systems rather than higher H_2_O_2_ levels, we performed the experiment with cells accumulating the cytosolic roGFP2-Orp1 sensor. This sensor barely reacts to H_2_O_2_ (Supplemental Figure S5, E and F) but is reduced by the same systems as roGFP2-Tsa2ΔC_R_. As shown in Supplemental Figure S8, the oxidation state of roGFP2-Orp1 did not increase under heat stress, leaving increased H_2_O_2_ concentrations as the source for roGFP2-Tsa2ΔC_R_ oxidation.

To test whether increased H_2_O_2_ levels under heat are derived from PET, we performed the experiment in the presence of DCMU. As the increase in H_2_O_2_ levels was the same in the presence of DCMU (Figure 5B), we concluded that H_2_O_2_ produced during heat stress does not derive from PET. Another possible source of H_2_O_2_ might be NAD(P)H oxidases at the plasma membrane, two of which are encoded by the *Chlamydomonas* genome: RESPIRATORY BURST OXIDASE 1 (RBO1, Cre03.g188300) and RBO2 (Cre03.g188400). To test their potential involvement, we performed the heat stress experiment with cells accumulating roGFP-Tsa2ΔC_R_ or roGFP2-Orp1 in the cytosol in the presence of the NAD(P)H oxidase inhibitor diphenyleneiodonium chloride (DPI). DPI led to an increased oxidation of both sensors under ambient and heat stress conditions, whereas it did not affect the increased oxidation of roGFP-Tsa2ΔC_R_ in response to heat (Supplemental Figure S8). Hence, DPI appears to impair cytosolic reducing systems in *Chlamydomonas* and does not allow conclusions about the source of H_2_O_2_ produced during heat stress.

We also applied the heat stress-recovery regime to cells accumulating the sensor in the stroma, the matrix, and the nucleus (Figure 5B). Heat stress led to a significant increase in H_2_O_2_ levels in the nucleus and a nonsignificant increase in the matrix; higher H_2_O_2_ levels in both compartments were abolished after recovery. In contrast, H_2_O_2_ levels in the stroma dropped slightly after heat stress, and significantly after the recovery period. These data point to a source of heat-induced H_2_O_2_ production outside of the chloroplast and mitochondria, and once again to limited intracellular diffusion of H_2_O_2_ concentrations.

## Discussion

### RoGFP2-Tsa2ΔC_R_, a sensor for the detection of H_2_O_2_ in five *Chlamydomonas* compartments

Here we report on the development of *Chlamydomonas* reporter strains accumulating the ultra-sensitive, genetically encoded H_2_O_2_ sensor roGFP2-Tsa2ΔC_R_ (Morgan et al., 2016) in six different subcellular compartments (cytosol, nucleus, ER, mitochondrial matrix, chloroplast stroma, and thylakoid lumen). The establishment of the sensor in *Chlamydomonas* was facilitated by using the MoClo technology (Weber et al., 2011) and the recently generated *Chlamydomonas* MoClo kit (Crozet et al., 2018). The interchangeability of individual parts and the ability to assemble them in a single reaction, combined with the short generation time of *Chlamydomonas*, enabled iterative cycles of construct production and testing in a very short time frame. Accordingly, problems encountered with insufficient promoter strength or inefficient targeting of the encoded sensor to the chloroplast and thylakoid lumen were rapidly solved.

We demonstrate the suitability of roGFP2-Tsa2ΔC_R_ to monitor intracellular H_2_O_2_ levels in *Chlamydomonas* with three different experimental setups, each having inherent advantages and limitations. First, we developed an assay using microtiter plates in a plate reader (Figures 2 and 3; Supplemental Figures S2C, S3, S4, and S5, E, and F). The advantage of this first setup is that changes in H_2_O_2_ levels can be monitored in real-time and at high throughput. Limitations of this assay are that cells need to be concentrated by centrifugation and are in the dark, which precludes the use of this approach to the monitoring of responses to changes in light intensity. In a second setup, we continuously circulated cells between culture flask and a cuvette in a spectrofluorometer (Figure 5A). The advantage of this setup is that changes in H_2_O_2_ levels can be monitored in real time under physiological conditions (if the dark period in the cuvette is not a problem), but this setup suffers from low throughput. In a third option, we trapped the sensor oxidation state with NEM (Figures 4 and 5B; Supplemental Figures S7 and S8). The advantage here is that measurements are under fully physiological conditions and at medium throughput. The limitation of NEM trapping is that H_2_O_2_ levels are not monitored in real time and therefore rapid H_2_O_2_ dynamics may be missed. The decision as to which setup to employ needs to be made depending on the specific biological question being asked.

### Properties of the roGFP2-Tsa2ΔC_R_ sensor in *Chlamydomonas* cells

Overall, the kinetics of oxidation and reduction of roGFP2-Tsa2ΔC_R_ after the exogenous addition of H_2_O_2_ were very similar to those observed for this sensor in yeast (Morgan et al., 2016) and for the roGFP2-Orp1 sensor in Arabidopsis seedlings (Ugalde et al., 2021). Consistent with previous observations in yeast, we observed that cytosolic roGFP2-Tsa2ΔC_R_ is >20% oxidized at steady state and that the oxidation of the untreated control sample decreases over time. In yeast, this decrease in sensor oxidation was shown to be caused by decreasing oxygen levels in the assay buffer (Morgan et al., 2016) and this is also likely the case in *Chlamydomonas* (Supplemental Figure S4). Thus, our observations indicate that, as in yeast, roGFP2-Tsa2ΔC_R_ functions as an ultra-sensitive H_2_O_2_ sensor in *Chlamydomonas* that enables the monitoring of “basal” cellular H_2_O_2_ levels. The exquisite sensitivity of the roGFP2-Tsa2ΔC_R_ in *Chlamydomonas* is reflected by the finding that upon the exogenous addition of H_2_O_2_, a roGFP2-Orp1 sensor accumulating in the *Chlamydomonas* cytosol to levels similar as the roGFP2-Tsa2ΔC_R_ sensor was barely responsive (Supplemental Figure S5). Accordingly, while 0.1 mM of exogenously added H_2_O_2_ induced a strong roGFP2-Tsa2ΔC_R_ response in *Chlamydomonas*, 2-mM H_2_O_2_ was required to elicit a roGFP2-Orp1 response in Arabidopsis seedlings, albeit a comparison of single cells with cells within the confine of tissues might not be entirely fair (Ugalde et al., 2021).

RoGFP2-Tsa2ΔC_R_ targeted to the ER was fully oxidized (Figure 2H). Full oxidation was also observed for an unfused roGFP2 targeted to the ER of tobacco (*Nicotiana tabacum*) leaf cells (Meyer et al., 2007) and for HyPer targeted to the ER in mammalian cells (Mehmeti et al., 2012). This is almost certainly due to the oxidative milieu in the ER lumen rather than to high ER lumenal H_2_O_2_ concentrations (Mehmeti et al., 2012). Specifically, the ER maintains an active disulfide-generating machinery and a relatively oxidized glutathione pool (in the absence of glutaredoxins) that does not allow sensor reduction (Schwarzlander et al., 2016). Hence, in this compartment disulfide bond formation is largely favored over disulfide reduction.

Cytosolic roGFP2 alone was insensitive to exogenously added H_2_O_2_ and was constantly in a highly reduced state (Figure 2C). RoGFP2 has been shown to be in equilibrium with the 2GSH/GSSG redox couple, which is catalyzed by enzymatically active glutaredoxins (Gutscher et al., 2008; Marty et al., 2009; Morgan et al., 2013). This points to a large excess of reduced glutathione (GSH) over oxidized glutathione (glutathione disulfide, GSSG) in the *Chlamydomonas* cytosol, as is the case for the cytosol of a wide variety of other cell types (Schwarzlander et al., 2016).

The expression of *HyPer* has been reported to be susceptible to gene silencing beyond the cotyledon stage in Arabidopsis (Exposito-Rodriguez et al., 2013). Although silencing can be circumvented, for example, by the transient expression in *N.* *benthamiana* abaxial epidermal cells (Exposito-Rodriguez et al., 2017), this is not ideal. We observed no silencing of our *roGFP2-Tsa2ΔC_R_* constructs over a period of 1–2 years in *Chlamydomonas*. However, although we employed all “tricks” for high-level transgene expression, which included using the strongest promoter currently available (*HSP70A-RBCS2*), a codon-optimized *ORF* containing the three *RBCS2* introns, the *RPL23* terminator, and the UVM4 expression strain (Schroda, 2019), sensor expression was only just sufficient to achieve a good signal-to-noise ratio in most compartments. When expression levels were reduced by about half, i.e., when the *PSAD* promoter was used instead of the *AR* promoter, or when the sensor was targeted to the thylakoid lumen, the signal became too noisy for reliable measurements, mainly because of the 405 nm channel (Supplemental Figure S3). Hence, for future applications of this sensor, care must be taken that accumulation levels of the sensor in the chosen *Chlamydomonas* strain are sufficiently high.

### H_2_O_2_ produced by PET in the stroma under physiological conditions readily diffuses into the cytosol but not to other subcellular compartments

Our observations demonstrated the suitability of roGFP2-Tsa2ΔC_R_ to monitor H_2_O_2_ levels under physiologically relevant conditions. For example, we observed a rapid, PET-dependent increase in stromal H_2_O_2_ levels under high light intensities (1,000 µmol photons m^−2^ s^−1^), together with a concomitant increase in cytosolic H_2_O_2_. Changes in H_2_O_2_ in the mitochondrial matrix were barely detectable and no change was observed in the nucleus. We also failed to detect an increase in H_2_O_2_ levels in the nucleus, even when we increased the light intensity to 1,500 µmol photons m^−2^ s^−1^ (Figure 4, D and E). However, we did detect H_2_O_2_ in the nucleus and mitochondria of cells supplemented with paraquat or metronidazole under low light intensities (Figure 4E). Thus, our findings partially corroborate earlier studies that reported leakage of PET-dependent H_2_O_2_ from the chloroplast into other compartments (Mubarakshina et al., 2010; Caplan et al., 2015; Exposito-Rodriguez et al., 2017; Ugalde et al., 2021). However, our results also indicate that the H_2_O_2_ scavenging capacity of the cytosol is sufficiently high to quench the comparably low H_2_O_2_ concentrations resulting from high light exposure, which effectively limits H_2_O_2_ diffusion into other subcellular compartments. In contrast, the cytosolic H_2_O_2_ scavenging capacity appears to become overwhelmed by the high H_2_O_2_ concentrations produced in the presence of metronidazole or paraquat (or exogenously added H_2_O_2_) and therefore H_2_O_2_ is not prevented from reaching other subcellular compartments.

In summary, our results strongly support the conclusion that cellular H_2_O_2_ scavenging enzymes limit the intracellular diffusion of H_2_O_2_, thereby leading to the establishment of steep intracellular H_2_O_2_ concentration gradients. This interpretation would be in line with the observation that efficient transfer of H_2_O_2_ from chloroplasts to the nucleus in *N.* *benthamiana* epidermal cells requires that the two compartments be in close proximity (Exposito-Rodriguez et al., 2017). This is possible because of the mobility of chloroplasts in land plant cells and appears unlikely for the architecture of *Chlamydomonas* cells with a single, large chloroplast.

### H_2_O_2_ is produced rapidly under heat stress and does not derive from PET

Cytosolic and nuclear H_2_O_2_ levels increased rapidly and transiently during heat stress (Figure 5). Rapid accumulation of H_2_O_2_ has been previously reported in tobacco seedlings, spinach (*Spinacia oleracea*) leaves, or Arabidopsis and tobacco cell cultures when exposed to heat stress and therefore appears to be a conserved response in plant cells (Foyer et al., 1997; Vacca et al., 2004; Volkov et al., 2006; Gómez et al., 2008). In *Chlamydomonas*, cytosolic H_2_O_2_ levels closely paralleled the temperature change in the culture, indicating that H_2_O_2_ appears to be derived from a constitutive temperature-dependent cellular process (Figure 5A). As the increase in cytosolic H_2_O_2_ levels was also observed in the presence of DCMU, it cannot derive from PET, a hypothesis that is confirmed by the observed decline of H_2_O_2_ levels in the stroma during heat stress and recovery (Figure 5B). The mild, transient increase of H_2_O_2_ levels in mitochondria also argues against this organelle as a source for heat-induced H_2_O_2_ production. However, there are many alternative production sites for H_2_O_2_ in plant cells that might be temperature-controlled, such as limited substrate oxidases (with glycolate, xanthin, urate, sulfite, mono-, and polyamines as substrates), type III peroxidases, or NAD(P)H oxidases at the plasma membrane (Cheeseman, 2007; Sun and Guo, 2016). Photorespiration is an unlikely source for H_2_O_2_ in *Chlamydomonas*, as it depends on input of electrons from PET and *Chlamydomonas* has a glycolate dehydrogenase that does not produce H_2_O_2_, rather than a glycolate oxidase (Kern et al., 2020).

Despite the rapid increase in H_2_O_2_ levels upon heat stress, *Chlamydomonas* cells accumulate scavenging enzymes such as superoxide dismutases, catalase, peroxiredoxins, or dehydroascorbate reductase only between 3 h and 24 h after the temperature shift (Mühlhaus et al., 2011; Hemme et al., 2014; Schroda et al., 2015). This observation indicates that the increased levels of H_2_O_2_ upon heat exposure are not detrimental to the cells during the first few hours at elevated temperatures and might play a role in signaling.

In summary, we report the generation of constructs enabling the accumulation of the ultra-sensitive H_2_O_2_ probe, roGFP2-Tsa2ΔC_R_, in six different subcellular compartments in *Chlamydomonas*. We show that these sensors respond readily to both exogenously added and endogenously produced H_2_O_2_ in five of these compartments and reveal that, by following the response of sensors targeted to multiple subcellular compartments, the existence of intracellular H_2_O_2_ gradients can be inferred. We anticipate that the future application of these sensors will allow exciting new insights into subcellular H_2_O_2_ homeostasis and dynamics.

## Materials and methods

### Strains and culture conditions

*Chlamydomonas reinhardtii* UVM4 cells (Neupert et al., 2009) were grown in Tris–Acetate–Phosphate (TAP) medium (Kropat et al., 2011) on a rotatory shaker at a constant light intensity of ∼30 μmol photons m^−2^ s^−1^ provided by MASTER LEDtube HF 1200 mm UO 16W830 T8 and 16W840 T8 (Philips). For high-light exposure, cells were grown to a density of ∼1×10^6^ cells·mL^−^^1^, transferred to an open 1-L beaker, placed on an orbital shaker, and exposed to 1,000 or 1,500 µmol photons m^−2^ s^−1^ provided by CF Grow (CXB3590-X4). Transformation was performed with the glass beads method (Kindle, 1990) as described previously (Hammel et al., 2020) with constructs linearized with NotI digestion. Transformants were selected on TAP medium containing 100-µg mL^−1^ spectinomycin.

### Cloning of sensor and signal peptide coding sequences

The roGFP2-Tsa2ΔC_R_ and roGFP2-Orp1 sequences (Gutscher et al., 2009; Morgan et al., 2016) were reverse-translated using the most highly used *Chlamydomonas* codons and equipped with the three *Chlamydomonas RBCS2* introns as previously suggested for foreign genes (Schroda, 2019), but using as much of the native flanking sites of these introns (CAA-Intron 1-GA, ACG-Intron 2-GC, and GC-Intron 3-CTG) as possible. For *roGFP2-Tsa2ΔC_R_*, an AAG Lys codon was converted into the suboptimal AAA Lys codon to eliminate a GAAGAC BpiI recognition site. The sequences were flanked with BsaI restriction sites such that upon BsaI digestion, fragments with AATG and TTCG overhangs are generated for the B3/4 position of level 0 parts according to the MoClo syntax for plant genes (Weber et al., 2011; Patron et al., 2015; Figure 1A). Synthesis and cloning into the pUC57 vector was accomplished by GeneCust (Luxembourg), giving rise to pMBS418 (*roGFP2-Orp1*) and pMBS419 (*roGFP2-Tsa2ΔC_R_*). The latter construct was used as template for the polymerase chain reaction (PCR) to amplify a 898-bp fragment containing only the *roGFP2* coding sequence with the first *RBCS2* intron. The PCR product was combined with the destination vector pAGM1287 (Weber et al., 2011), digested with BpiI and assembled by ligation into the level 0 construct pMBS467. The level 0 part encoding the HSP70B chloroplast transit peptide was generated via PCR using plasmid pMBS197 as template. This plasmid harbors the sequence encoding the HSP70B transit peptide with an intron, in which a BsaI recognition site was eliminated. The resulting 251-bp product and plasmid pAGM1276 (Weber et al., 2011) were digested with BpiI and ligated to yield pMBS639. The level 0 part for the chloroplast transit peptide of CDJ1 was similarly produced by PCR using genomic DNA as template, generating a 166-bp product and giving rise to pMBS640. The same procedure was followed to produce level 0 parts for bipartite transit peptides for stroma and thylakoid lumen of PSAN and PSBO, to generate PCR products of 508 bp and 470 bp, giving rise to pMBS298 (PSAN) and pMBS641 (PSBO), respectively. The primers used are listed in Supplemental Table S1. All PCRs were conducted with Q5 High-Fidelity Polymerase (NEB) following the manufacturer’s instructions. Error-free cloning was verified by Sanger sequencing. These newly constructed level 0 parts were then complemented with level 0 parts (pCM) from the *Chlamydomonas* MoClo toolkit (Crozet et al., 2018) to fill the respective positions in level 1 modules as follows: A1-B1 – pCM0-015 (*HSP70A-RBCS2* promoter + 5′-untranslated region (UTR)); A1-B2 – pCM0-020 (*HSP70A-RBCS2* promoter + 5′-UTR); or pCM0-016 (*PSAD* promoter + 5′-UTR); B2 – pCM0-053 (USPA chloroplast transit peptide), pMBS639 (HSP70B chloroplast transit peptide), pMBS640 (CDJ1 chloroplast transit peptide), pMBS298 (PSAN thylakoid lumen targeting peptide), pMBS641 (PSBO thylakoid lumen targeting peptide), pCM0-057 (HSP70C mitochondrial transit peptide), or pCM0-056 (BIP1 ER targeting signal); B3/4 – pMBS419 (roGFP2-Tsa2ΔC_R_), pMBS418 (roGFP2-Orp1), or pMBS467 (roGFP2); B5 – pCM0-100 (3xHA), pCM0-101 (MultiStop), pCM-111 (BIP ER retention sequence), or pCM-109 (SV40 nuclear localization signal); B6 – pCM0-119 (*RPL23* 3′-UTR). The *HSP70A-RBCS2* fusion promoter used here contains 467 bp of *HSP70A* sequences upstream of the start codon in optimal spacing with respect to the *RBCS2* promoter (Lodha et al., 2008; Strenkert et al., 2013). The respective level 0 parts and destination vector pICH47742 (Weber et al., 2011) were combined with BsaI and T4 DNA ligase and directionally assembled into the 11 level 1 modules shown in Figure 1B. The level 1 modules were then combined with pCM1-01 (level 1 module with the *aadA* gene conferring resistance to spectinomycin flanked by the *PSAD* promoter and terminator) from the *Chlamydomonas* MoClo kit, with plasmid pICH41744 containing the proper end-linker, and with destination vector pAGM4673 (Weber et al., 2011), digested with BpiI, and ligated to yield the 11 level 2 devices displayed in Figure 1B. All MoClo constructs employed and generated are listed in Supplemental Table S2. All newly generated plasmids can be ordered from the *Chlamydomonas* Research Center (https://www.chlamycollection.org/).

### Protein analysis by SDS–PAGE

Protein extraction (SDS–PAGE, semi-dry blotting and immunodetections were carried out as described previously (Hammel et al., 2020). Primary antibodies used for immunodetection were mouse anti-HA (Sigma H9658, 1:10,000) and rabbit anti-GFP (Roche, Cat. No. 11814460001, 1:5,000). The secondary antibody was m-IgGκBP-HRP (Santa Cruz Biotech sc-516102, 1:10,000). Densitometric band quantifications after immunodetections were done with the FUSIONCapt Advance program (PEQLAB).

### RoGFP2 fluorescence recordings

RoGFP2 fluorescence was recorded using a CLARIOstar fluorescence plate reader (BMG-Labtech) as described previously for yeast (Morgan et al., 2016) and adapted to *Chlamydomonas* as follows: *Chlamydomonas* cells were grown in constant light to a density of ∼3×10^6^ cells·mL^−^^1^. 1×10^7^ cells were used per well and were harvested by centrifugation at 3,150*g* for 2 min at 25°C. Cells were resuspended in 100-mM MES–Tris buffer (pH 7.0) to a cell density of 5×10^7^ cells·mL^-1^; 200 μL of cell suspension was transferred to each well of a black, clear flat-bottomed 96-well imaging plate (BD Falcon 353219). For calibration and later data processing, control samples of fully oxidized and fully reduced sensors were prepared by adding N,N,N′,N′-tetramethylazodicarboxamide (diamide) to a final concentration of 20 mM and DTT to a final concentration of 100 mM to cells in two different wells, respectively. Additional wells to record roGFP2 fluorescence at steady state, at increasing H_2_O_2_ concentrations, or after different treatments were prepared. Each biological replicate was measured as technical duplicates. Before starting the measurement, the 96-well imaging plate was centrifuged at 30*g* for 5 min at 25°C. *Chlamydomonas* UVM4 recipient cells were always included as negative control. The measurement mode of the plate reader was based on the “Fluorescence Intensity” coupled with the “Bottom Optic” option, with a positioning delay of 0.2 s, 40 flashes per well and the desired number of measurement cycles (about 200 cycles) and time per cycle (about 20 s). The gain adjustment was set to 80% maximum and an automatic gain adjustment for each wavelength was performed immediately before the measurement. RoGFP2 exhibits two excitation maxima at 400 nm and 475–490 nm with fluorescence emission monitored at 510 nm. Therefore, we ensured that the number of multichromatics was set to 2 and that the correct excitation (410 and 480 nm) and emission (510 nm) filters were selected. The measurement was started and increasing H_2_O_2_ solutions were added after 10 cycles. After the fluorescence measurement was done, BMG Labtech MARS Data Analysis software was used to subtract the auto-fluorescence of UVM4 control cells. The “blank corrected” values were taken to calculate the degree of sensor oxidation (OxD) using the following equation (Meyer and Dick, 2010):

$$\mathrm{OxD}\mathrm{roGFP}2\frac{{I405}_{\mathrm{sample}}\times {I488}_{\mathrm{red}}-{I405}_{\mathrm{red}}\times {I488}_{\mathrm{sample}}}{{I405}_{\mathrm{sample}}\times {I488}_{\mathrm{red}}-{I405}_{\mathrm{sample}}\times {I488}_{\mathrm{ox}}+{I405}_{\mathrm{ox}}\times {I488}_{\mathrm{sample}}-{I405}_{\mathrm{red}}\times {I488}_{\mathrm{sample}}}$$

where *I* is the fluorescence intensity at 510 nm after excitation at either 405 nm or 488 nm. The subscripts “ox”, “red”, and “sample” indicate the fluorescence intensity measured for the fully oxidized and fully reduced controls and the sample, respectively.

### NEM-based alkylation for measuring H_2_O_2_ levels in cell cultures.

The exact cell number of *Chlamydomonas* cells grown to 1–3×10^6^ cells·mL^−^^1^ was determined and the volume to harvest 2.5×10^7^ cells was calculated. For rapid “trapping” of probe oxidation, 50-mL Falcon tubes were preloaded with one fourth of the final volume with 100-mM MES–Tris (pH 7.0) buffer containing 40-mM NEM. *Chlamydomonas* cells were harvested in prefilled Falcon tubes by centrifugation at 3,150*g* for 3 min at room temperature and subsequently resuspended in 500 µL of 100-mM MES–Tris (pH 7.0) buffer containing 40-mM NEM to a final cell density of 5×10^7^ cells·mL^−^^1^. 200 µL (corresponding to 10^7^ cells) of cell suspension was transferred to a 96-well plate. For calculating OxD, fully oxidized (with diamide) and reduced (with DTT) samples were added and probe oxidation was measured following the protocol described above with 15 measurements per biological sample to reduce technical variability. To verify efficient trapping of the sensor oxidation state with NEM, roGFP2 fluorescence recordings using 0.1-mM H_2_O_2_ were performed as described above. Twenty microliters of NEM (200-mM stock) was added to a final concentration of 16.67-mM NEM per well at the indicated time differences.

### Continuous monitoring of real-time changes in H_2_O_2_ levels during heat stress via connected tubing and a peristaltic pump

*Chlamydomonas* cells (∼5×10^6^ cells/mL) were cultivated under agitation at a constant light intensity of ∼30 μmol photons m^**−**^^2^ s^**−**^^1^ and continuously circulated through a quartz cuvette inserted into a spectrofluorometer (Jasco FP-8300) via connected tubing and a peristaltic pump (Watson Marlow 101U, flow rate: 1 mL·s^−^^1^). After 20 min, cells were exposed to 40°C for 30 min in a water bath, and shifted back to 23°C for 30 min. The ratio of the fluorescent light intensities emitted after excitation at 405 and 488 nm was monitored every 30 s. The measurement mode of the spectrofluorometer was based on the “Fixed wavelength measurement” with an excitation bandwidth of 5 nm and an emission bandwidth of 10 nm.

### Confocal microscopy

All images were acquired using a Zeiss LSM880 AxioObserver confocal laser scanning microscope equipped with a Zeiss C-Apochromat 40x/1.2 W AutoCorr M27 water-immersion objective. Fluorescent signals of GFP (excitation/emission 488 nm/491–589 nm) and chlorophyll autofluorescence (excitation/emission 633 nm/647–721 nm) were processed using the Zeiss software ZEN 2.3 or ImageJ.

## Supplemental data

The following materials are available in the online version of this article.

**Supplemental Figure S1**. Screening of transformants accumulating the roGFP2 sensor by immunoblotting.

**Supplemental Figure S2**. Localization and fluorescence properties of a weakly accumulating, stroma-targeted roGFP2-Tsa2ΔC_R_ sensor.

**Supplemental Figure S3**. Fluorescence readout at excitation wavelengths 405 nm and 488 nm in the best accumulating *ARpro* and *PSADpro* transformants.

**Supplemental Figure S4**. Real-time monitoring of H_2_O_2_ levels in cytosol and stroma under steady-state conditions at different cell densities.

**Supplemental Figure S5**. Establishment of a cytosolic roGFP2-Orp1 sensor.

**Supplemental Figure S6**. Effect of metronidazole and paraquat on growth.

**Supplemental Figure S7**. Analysis of fluorescence properties of the roGFP2-Tsa2ΔC*_R_* sensor at different temperatures.

**Supplemental Figure S8**. Analysis of the effects of the NAD(P)H oxidase inhibitor diphenyleneiodonium chloride (DPI) and heat on sensor oxidation.

**Supplemental Table S1**. Primers used for cloning.

**Supplemental Table S2**. MoClo constructs employed and generated.

**Supplemental Table S3**. Transgenic lines generated, number of transformants analyzed, and number of localizations observed in different cells.

**Supplemental Data Set S1**. Summary of statistical analyses.

## Supplementary Material

koab176_Supplementary_Data

## Notes

J.N. generated all constructs and performed all the experiments. D.S. recorded the confocal microscopy images. J.O. helped with setting up the fluorescence recordings. M.S. and B.M. conceived and supervised the project and wrote the article with contributions from all the authors.

The author responsible for distribution of materials integral to the findings presented in this article in accordance with the policy described in the Instructions for Authors (https://academic.oup.com/plcell) is: Michael Schroda (schroda@bio.uni-kl.de).
