# Supplementary material for: Real-time monitoring of subcellular H2O2 distribution in Chlamydomonas reinhardtii
Source: Plant Cell. 2021 Jul 1;33(9):2935–49. doi: 10.1093/plcell/koab176 (PMC8462822; doi:10.1093/plcell/koab176)
Supplement: koab176_Supplementary_Data [file koab176_supplementary_data.zip › tpc.00945.2020-s01.pdf]

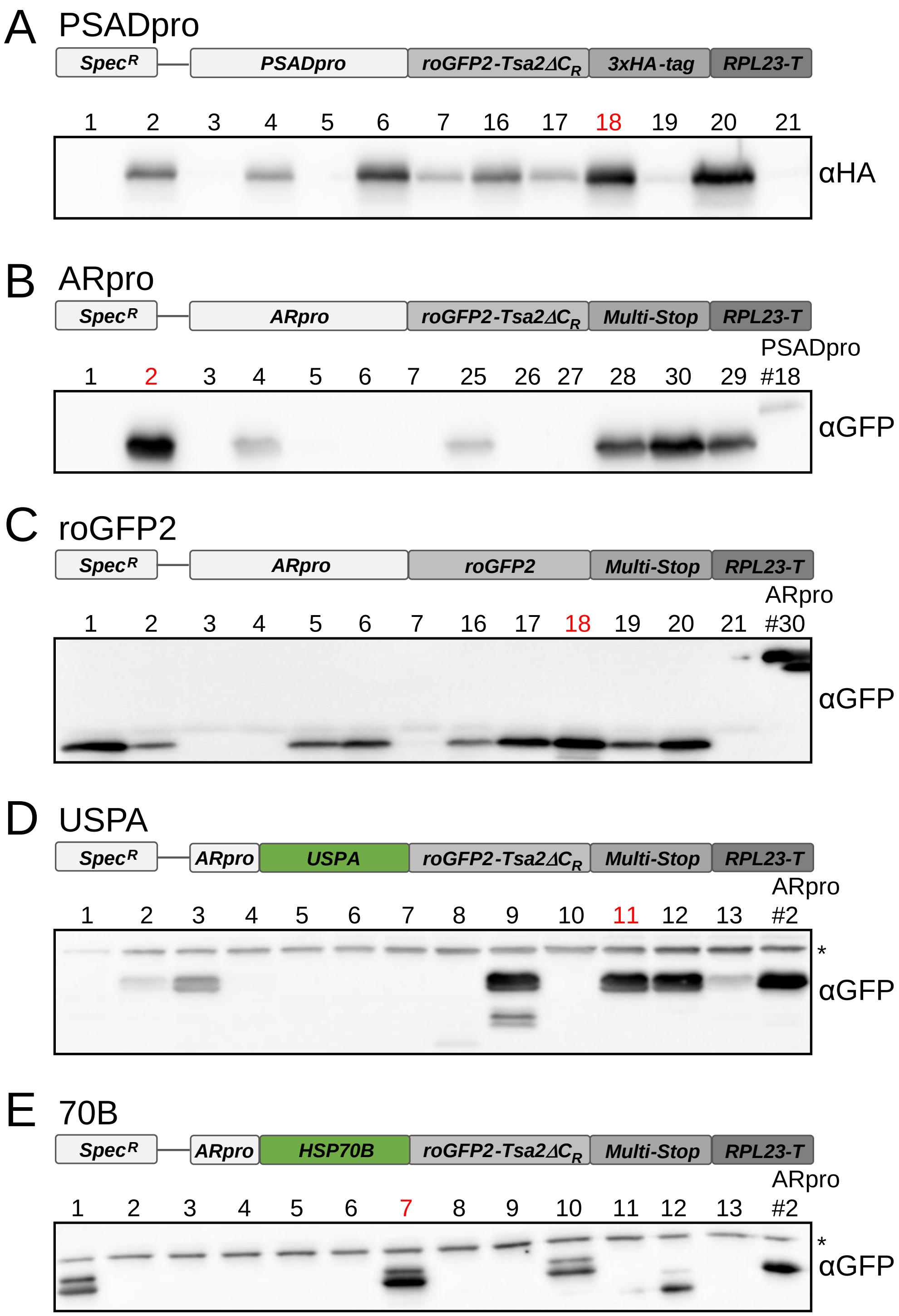

Supplemental Figure S1. Screening of transformants accumulating the roGFP2 sensor by immunoblotting.
(A-K) Total cell protein extracts corresponding to 1.5 µg chlorophyll for each transformant were separated by SDS-PAGE and analyzed by immunoblotting using an antibody against GFP or the HA epitope. The transformant number is given on top of each panel. The number in red indicates the transformant chosen for further analysis. The asterisk indicates a protein band cross-reacting with the GFP antibody.
Supports Figure 1.

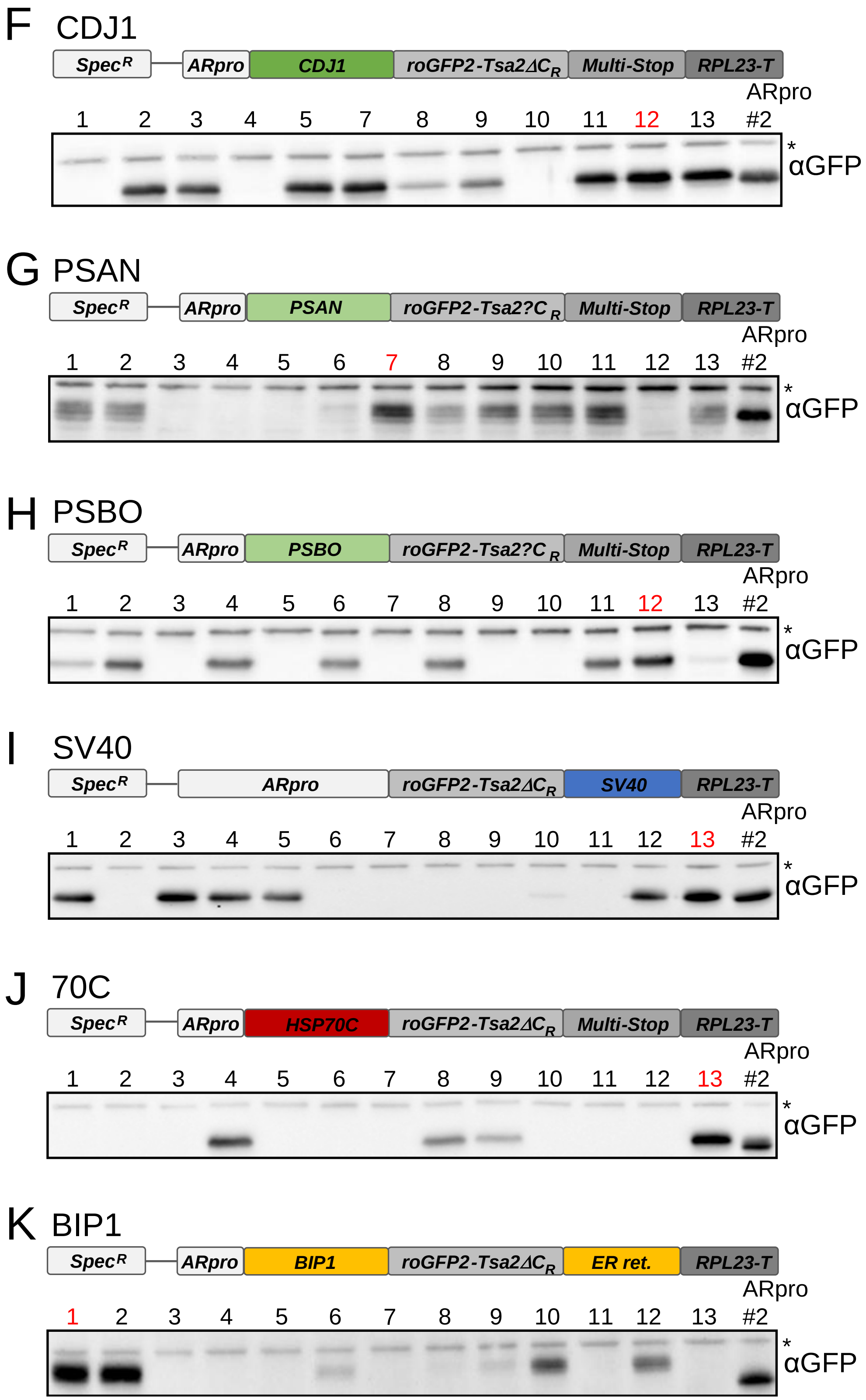

Supplemental Figure S1, continued.

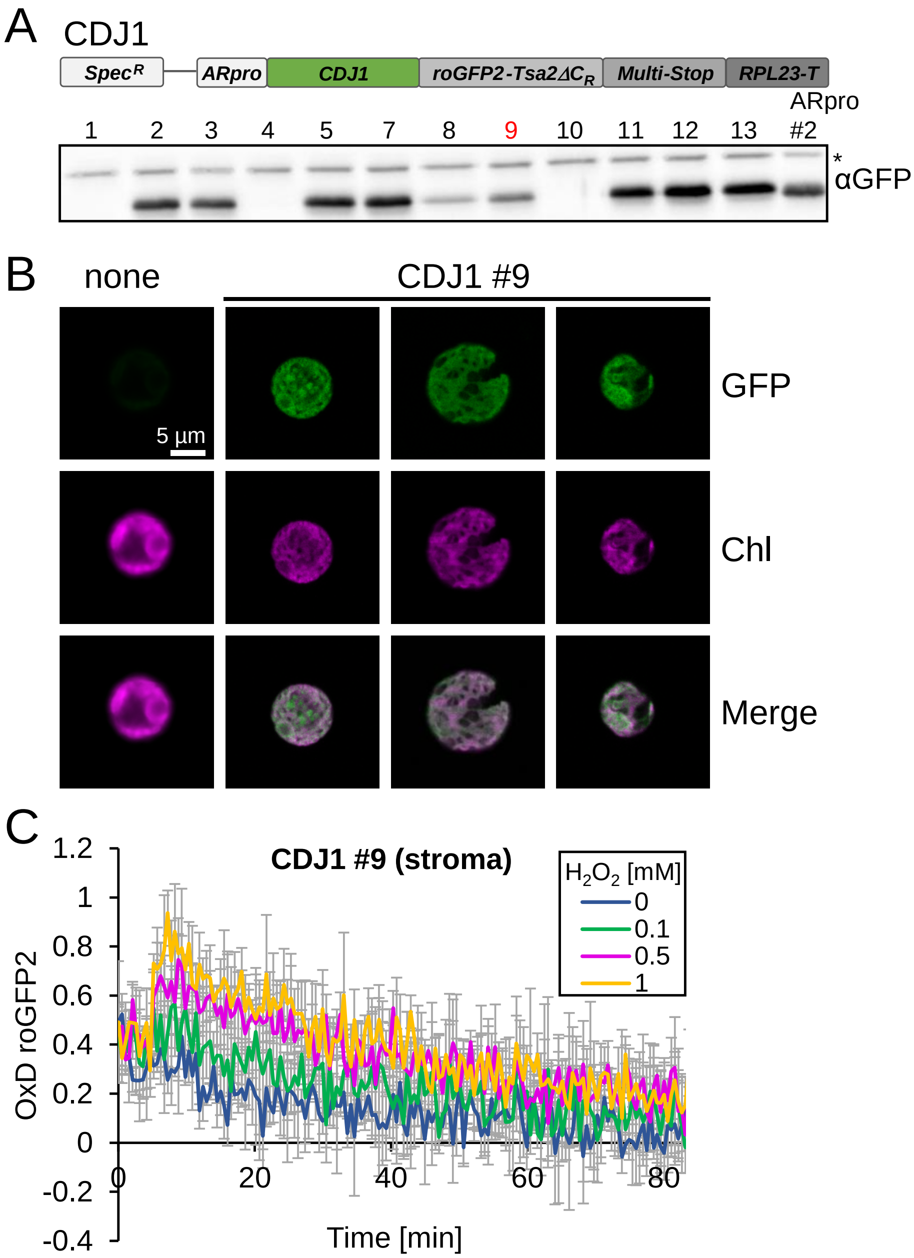

Supplemental Figure S2. Localization and fluorescence properties of a weakly accumulating, stroma-targeted roGFP2-Tsa2ΔC$_R$ sensor.

(A) Comparison of sensor accumulation levels across CDJ1 transformants (reproduced from Supplemental Figure S1F).

(B) Confocal microscopy images of individual cells of the weakly accumulating transformant #9 shown in (A) and the untransformed UVM4 strain (none). Shown are GFP fluorescence, chlorophyll autofluorescence (Chl) and both signals merged.

(C) Fluorescence measurement of roGFP2-Tsa2ΔC$_R$ in transformant #9 under steady-state conditions (no H_2O_2 added, blue) and after the addition of H_2O_2 at concentrations of 0.1 mM (green), 0.5 mM (magenta), and 1 mM (yellow). Values were calculated relative to those obtained for fully reduced (0) and fully oxidized (1) sensors. Shown are means from three independent experiments. Error bars represent standard deviation.

Supports Figures 1 and 2.

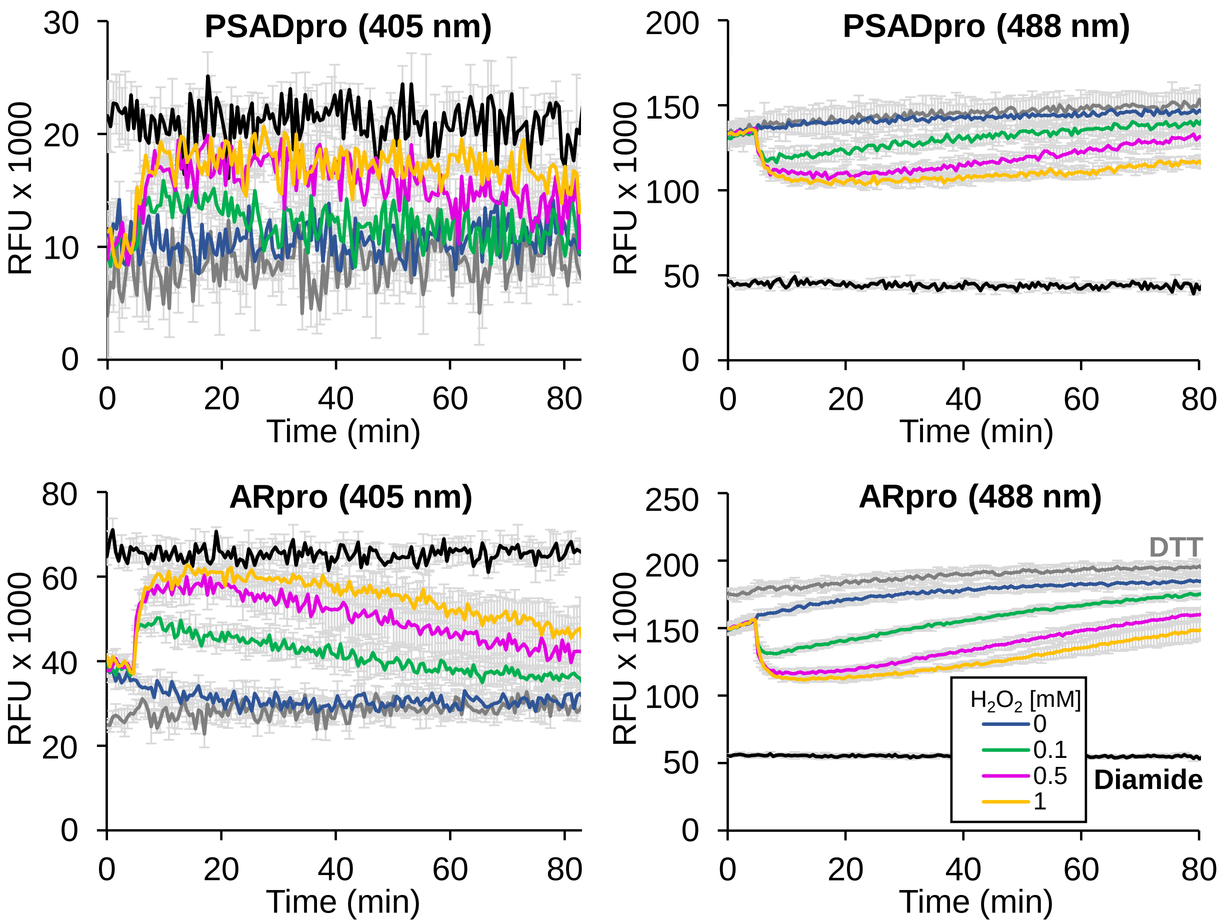

Supplemental Figure S3. Fluorescence readout at excitation wavelengths 405 nm and 488 nm in the best accumulating *ARpro* and *PSADpro* transformants.
Fluorescence measurement at 405 nm (left panels) and 488 nm (right panels) of cytosolic roGFP2-Tsa2ΔC$_R$ in the best accumulating transformants with the *PSAD* (*PSADpro*) or *AR* (*ARpro*) promoters. Full sensor reduction was achieved by the addition of 100 mM DTT (gray) and full sensor oxidation by the addition of 20 mM diamide (black). Cells were kept under steady-state conditions (no H_2O_2 added, blue) or treated with H_2O_2 at concentrations of 0.1 mM (green), 0.5 mM (magenta), and 1 mM (yellow). Shown are data from three independent experiments. Error bars represent standard deviation. Supports Figure 2.

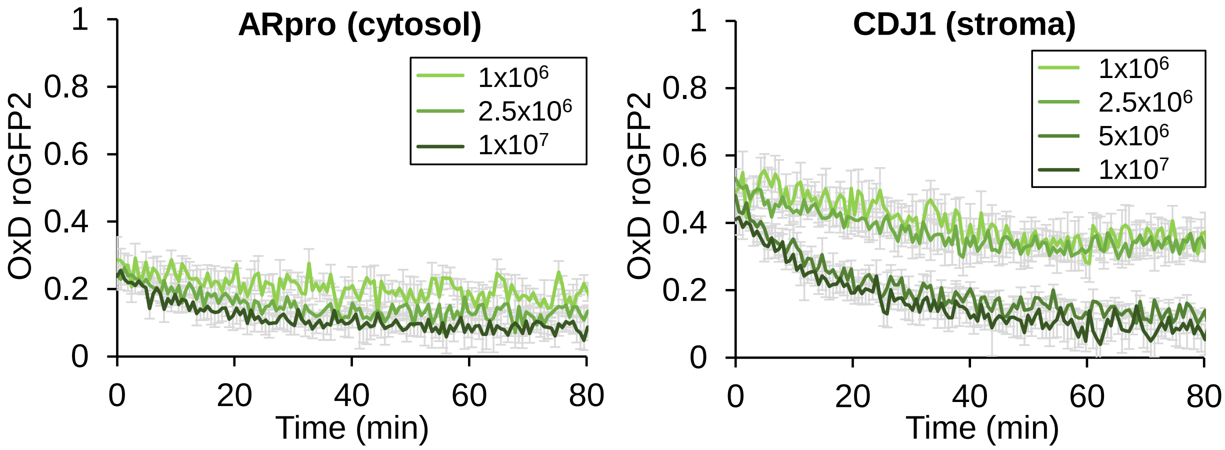

Supplemental Figure S4. Real-time monitoring of H_2O_2 levels in cytosol and stroma under steady-state conditions at different cell densities.
Fluorescence measurement of roGFP2-Tsa2ΔC$_R$ in cytosol and stroma under steady-state conditions with the cell densities indicated. Values were calculated relative to those obtained for fully reduced (0) and fully oxidized (1) sensors. Shown are means from three independent experiments. Error bars represent standard deviation. Supports Figure 2.

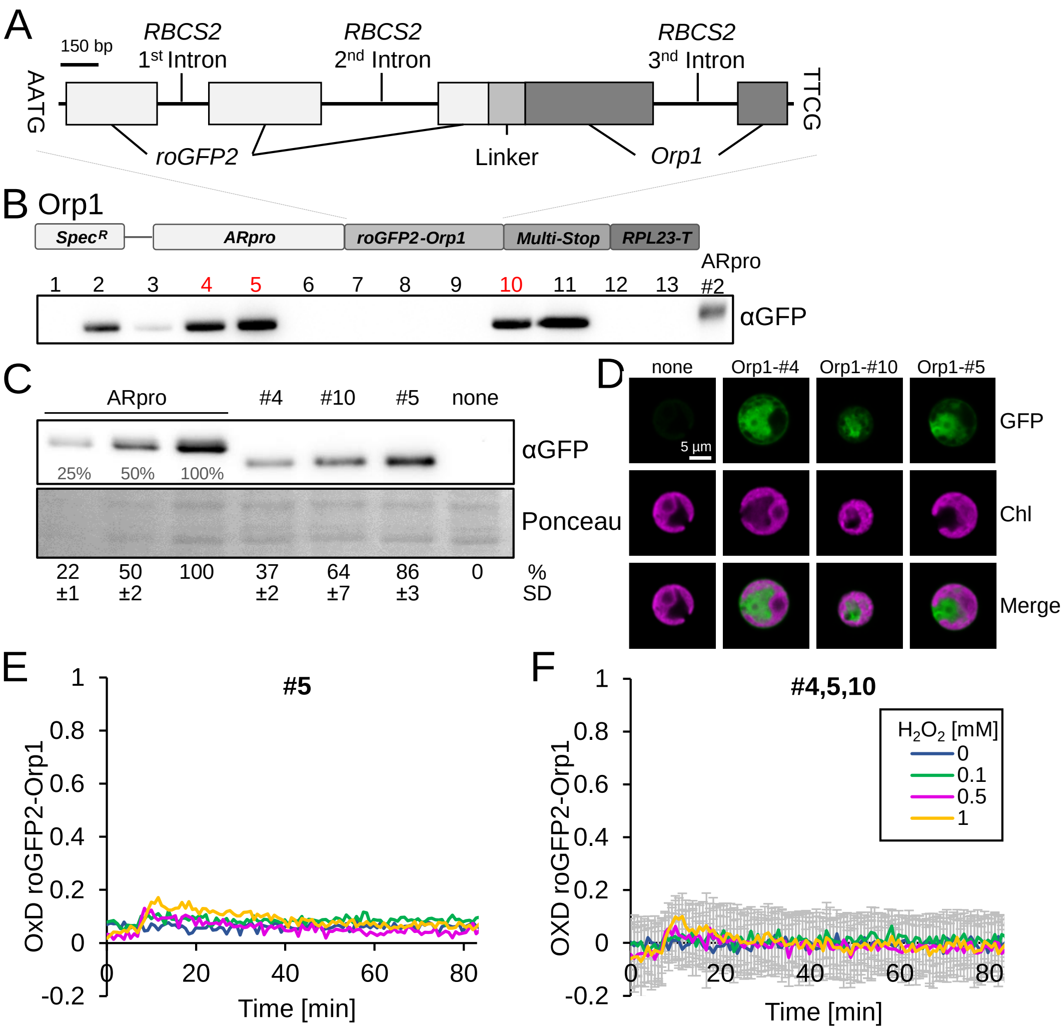

Supplemental Figure S5. Establishment of a cytosolic roGFP2-Orp1 sensor.
(A) Level 0 part encoding the roGFP2-Orp1 H_2O_2 sensor. The coding region (exons shown as boxes), interrupted by the three *RBCS2* introns (thin lines), was synthesized with optimal Chlamydomonas codon usage. RoGFP2 (light gray) is separated from Orp1 (dark gray) by a linker (gray).
(B) Level 2 construct and screening of transformants accumulating roGFP2-Orp1 by immunoblotting analysis, as described in Supplemental Figure S1.
(C) Comparison of the highest-accumulating roGFP2-Orp1 transformants from (B) with the highest-accumulating transformant targeting roGFP2-Tsa2ΔC$_R$ to the cytosol (ARpro). Total cell protein extracts were analyzed by immunoblotting using an antibody against GFP as described in Figure 1. The UVM4 recipient strain (none) served as negative control. GFP signals from three independent experiments were quantified and normalized to the signal obtained with the ARpro transformant. Mean values are given below the panel (± standard deviation). A representative experiment is shown, with Ponceau staining demonstrating equal loading.
(D) Representative confocal microscopy images of individual cells of the highest-accumulating transformants shown in (C) and the untransformed UVM4 strain (none). Shown are GFP fluorescence, chlorophyll autofluorescence (Chl) and both signals merged.
(E, F) Fluorescence measurement of roGFP2-Orp1 under steady-state conditions (no H_2O_2 added, blue) and after the addition of H_2O_2 at concentrations of 0.1 mM (green), 0.5 mM (magenta), and 1 mM (yellow). Values were calculated relative to those obtained for fully reduced (0) and fully oxidized (1) sensors. Shown are data for the best expressing transformant #5 (E) and means of measurements on transformant #4, #5, and #10 (F). Error bars represent standard deviation. Supports Figure 2.

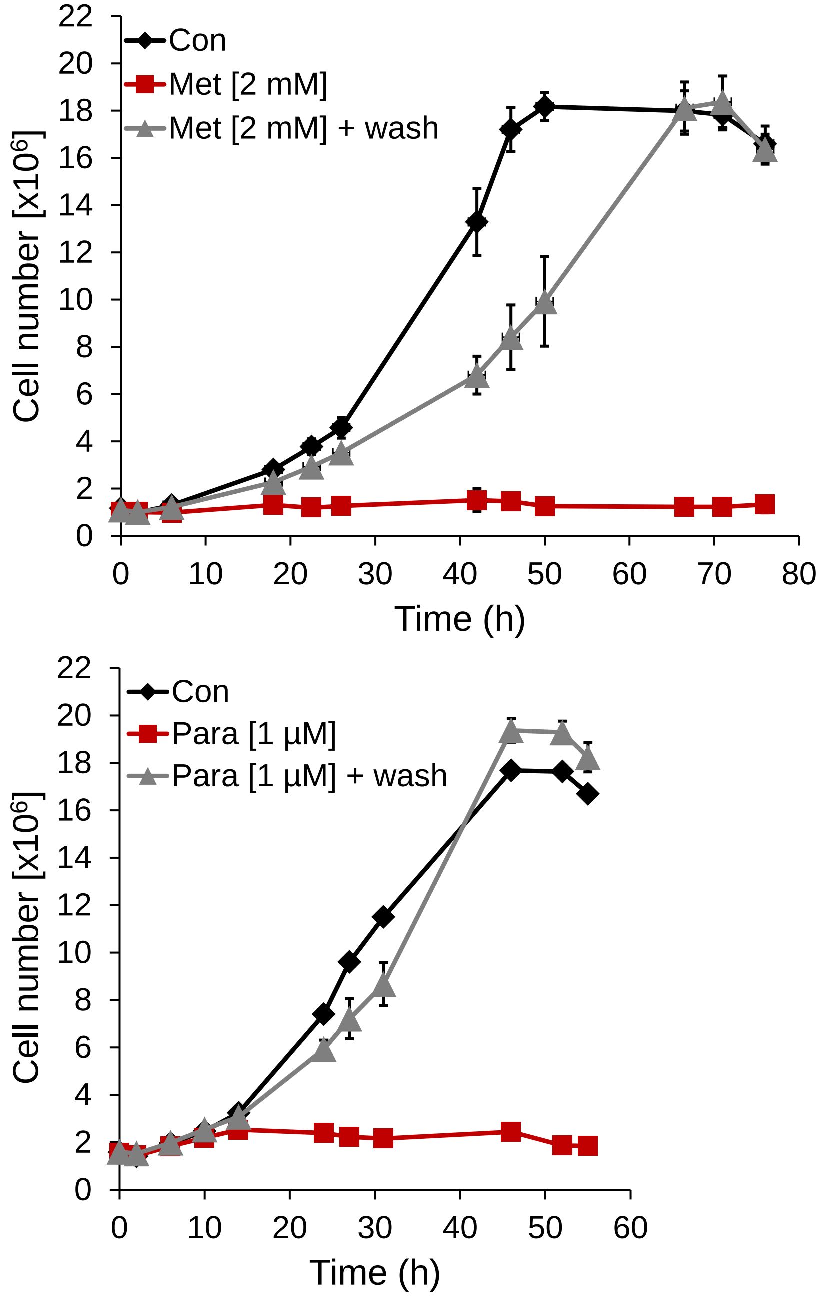

Supplemental Figure S6. Effect of metronidazole and paraquat on growth.
Two batches of transformant cells accumulating the roGFP2-Tsa2ΔC$_R$ sensor in the cytosol were grown in TAP medium in a low light intensity of ~60 µmol photons m^{-2} s^{-1} at 23°C. Cells were diluted and split into two x three cultures.
(A) Two cultures from one batch were treated with 2 mM metronidazole for 1 h, the third culture was left untreated (Con). Cells in all cultures were centrifuged, washed, and resuspended in TAP medium. To one of the metronidazol-treated cultures, the drug was added again (Met), while it was not added again to the second (Met + wash). Cell numbers were recorded over 78 h, shown are mean values from three independent experiments, error bars are standard deviation.
(B) Same procedure as described in (A) with 1 µM paraquat (Para) instead of metronidazol.
Supports Figure 4.

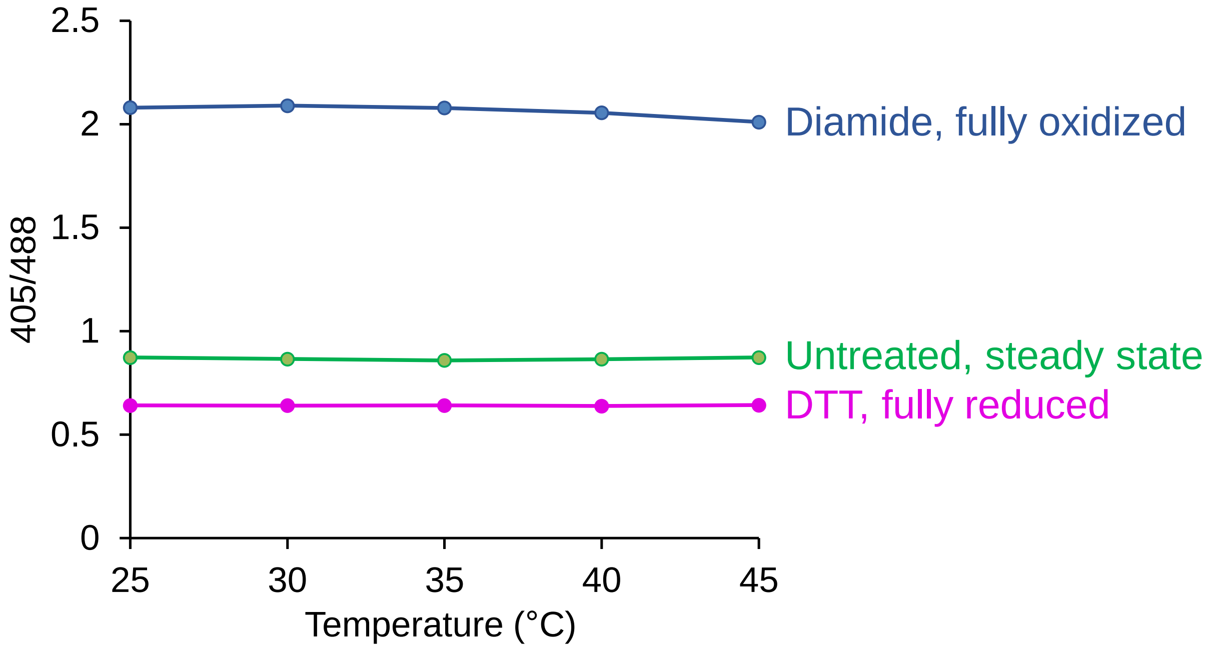

Supplemental Figure S7. Analysis of fluorescence properties of the roGFP2- Tsa2ΔC_R sensor at different temperatures.
Transformant cells accumulating the sensor in the cytosol were grown in a low light intensity of 30 μmol photons m^{-2} s^{-1} at 23°C. Cells were incubated with 20 mM diamide or 10 mM DTT to fully oxidize or reduce the sensor, respectively, or left untreated to leave the sensor in steady-state (SS). The oxidation state of the sensor was trapped by the addition of NEM. The temperature of the plate was set to the indicated temperature and GFP fluorescence measured in a plate reader after excitation at 405 and 488 nm, respectively.
Supports Figure 5.

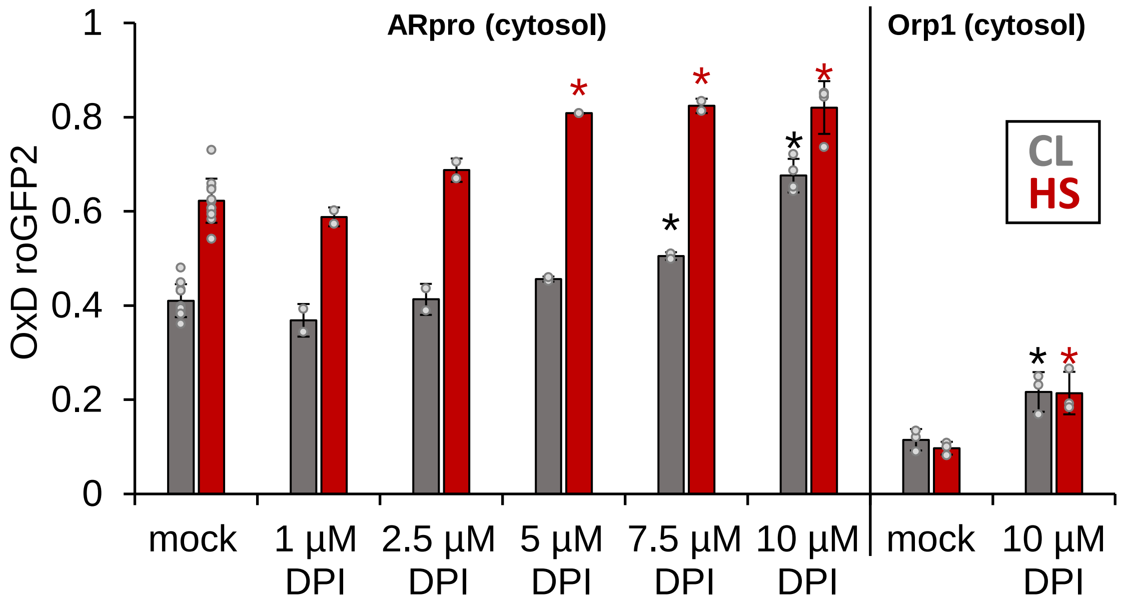

Supplemental Figure S8. Analysis of the effects of the NAD(P)H oxidase inhibitor diphenyleneiodonium chloride (DPI) and heat on sensor oxidation.
Transformant cells accumulating the sensors roGFP2-Tsa2ΔCR (ARpro) or roGFP2-Orp1 (Orp1) in the cytosol were grown in low light of 30 µmol photons m^{-2} s^{-1} at 23°C and then exposed to 40°C for 30 min (HS) or left at 23°C (CL), Cultures were treated with the indicated final concentrations of diphenyleneiodonium chloride (DPI) before the experiment was started. The oxidation state of the sensor was trapped by the addition of NEM and roGFP2 fluorescence was measured in a plate reader. Error bars represent standard deviation from 2-4 independent experiments for DPI treatments and mock- treated Orp1, and from 12 independent experiments for mock-treated ARpro. Asterisks indicate significant differences with respect to the mock control under nonstress (gray, CL) and heat stress (red, HS) conditions (two-tailed, unpaired *t*- test with Bonferroni-Holm correction, $P < 0.05$). The absence of an asterisk means that there were no significant differences.
Supports Figure 5.

Supplemental Table S1. Primers used for cloning.

Primer	Sequence	Target
roGFP2-for	5'-TTGAAGACATAATGGCCAGCGAGTTCAGC-3'	*roGFP2*
roGFP2-rev	5'-TTGAAGACTCCGAACCCTTGTACAGCTCGTCCATGCC-3'	
CTP-BirA-1	5'-TTGAAGACAACCATGCCGGTTCAGCAGATGAC-3'	HSP70B chloroplast transit peptide
CTP-BirA-2D	5'-TTGAAGACAACATTGCCTGAAGGAACAATTCAAATG-3'	
CTCDJ1-1	5'-TTGAAGACAACCATGCTCGCAAACCTTCGTAG-3'	CDJ1 chloroplast transit peptide
CTCDJ1-2	5'-TTGAAGACAACATtCCGTCGGCCCGCACAACCAC-3'	
PSAN-cpTAT-1	5'-AAGAAGACCACCATGGCCATCTCTGCTCGCTC-3'	PSAN thylakoid lumen transit peptide
PSAN-cpTAT-2	5'-TTGAAGACGGCATTCCGGCGTTGGCGACGGGGGC-3'	
TPPSBO-1	5'-TTGAAGACAACCATGGCCCTCCGCGCTGCCCA-3'	PSBO thylakoid lumen transit peptide
TPPSBO-2	5'-TTGAAGACAACATTGCgAGGGCGTTGGCCGACTGCGA-3'	

BpiI recognition sites are underlined

Supplemental Table S2. MoClo constructs employed and generated.

Plasmid	Description	Level	Source
pCM0-015	*HSP70A-RBCS2* promoter + 5'UTR	0	Crozet et al., 2018
pCM0-020	*HSP70A-RBCS2* promoter + 5'UTR	0	Crozet et al., 2018
pCM0-016	*PSAD* promoter + 5' UTR	0	Crozet et al., 2018
pCM0-053	USPA chloroplast transit peptide	0	Crozet et al., 2018
pCM0-057	HSP70C mitochondrial transit peptide	0	Crozet et al., 2018
pCM0-056	BIP1 ER targeting signal	0	Crozet et al., 2018
pCM0-100	3xHA	0	Crozet et al., 2018
pCM0-101	MultiStop	0	Crozet et al., 2018
pCM-111	BIP ER retention sequence	0	Crozet et al., 2018
pCM-109	SV40 nuclear localization signal	0	Crozet et al., 2018
pCM0-119	*RPL23* 3' UTR	0	Crozet et al., 2018
pMBS639	HSP70B chloroplast transit peptide	0	This study
pMBS640	CDJ1 chloroplast transit peptide	0	This study
pMBS298	PSAN thylakoid lumen targeting peptide	0	This study

pMBS641	PSBO thylakoid lumen targeting peptide	0	This study
pMBS418	*roGFP2-Orp1*	0	This study
pMBS419	*roGFP2-Tsa2ΔC_R*	0	This study
pMBS467	*roGFP2*	0	This study
pCM1-01	*PSADpro::aadA::PSAD-T*	1	Crozet et al., 2018
pMBS469	*ARpro::roGFP2-Tsa2ΔC_R::MStop::RPL23-T*	1	This study
pMBS425	*PSADpro::roGFP2-Tsa2ΔC_R::3xHA::RPL23-T*	1	This study
pMBS642	*ARpro::USPA::roGFP2-Tsa2ΔC_R::MStop::RPL23-T*	1	This study
pMBS643	*ARpro::HSP70B::roGFP2-Tsa2ΔC_R::MStop::RPL23-T*	1	This study
pMBS644	*ARpro::CDJ1::roGFP2-Tsa2ΔC_R::MStop::RPL23-T*	1	This study
pMBS645	*ARpro::PSAN::roGFP2-Tsa2ΔC_R::MStop::RPL23-T*	1	This study
pMBS646	*ARpro::PSBO::roGFP2-Tsa2ΔC_R::MStop::RPL23-T*	1	This study
pMBS649	*ARpro::roGFP2-Tsa2ΔC_R::SV40::RPL23-T*	1	This study
pMBS647	*ARpro::HSP70C::roGFP2-Tsa2ΔC_R::MStop::RPL23-T*	1	This study
pMBS648	*ARpro::BIP1::roGFP2-Tsa2ΔC_R::ER-ret::RPL23-T*	1	This study
pMBS470	*ARpro::roGFP2::MStop::RPL23-T*	1	This study
pMBS719	*ARpro::roGFP2-Orp1::MStop::RPL23-T*	1	This study
pMBS472	*PSADpro::aadA::PSAD-T::::*<br>*ARpro::roGFP2-Tsa2ΔC_R::MStop::RPL23-T*	2	This study
pMBS431	*PSADpro::aadA::PSAD-T::::*<br>*PSADpro::roGFP2-Tsa2ΔC_R::3xHA::RPL23-T*	2	This study
pMBS650	*PSADpro::aadA::PSAD-T::::*<br>*ARpro::USPA::roGFP2-Tsa2ΔC_R::MStop::RPL23-T*	2	This study
pMBS651	*PSADpro::aadA::PSAD-T::::*<br>*ARpro::HSP70B::roGFP2-Tsa2ΔC_R::MStop::RPL23-T*	2	This study
pMBS652	*PSADpro::aadA::PSAD-T::::*<br>*ARpro::CDJ1::roGFP2-Tsa2ΔC_R::MStop::RPL23-T*	2	This study
pMBS653	*PSADpro::aadA::PSAD-T::::*<br>*ARpro::PSAN::roGFP2-Tsa2ΔC_R::MStop::RPL23-T*	2	This study
pMBS654	*PSADpro::aadA::PSAD-T::::*<br>*ARpro::PSBO::roGFP2-Tsa2ΔC_R::MStop::RPL23-T*	2	This study
pMBS657	*PSADpro::aadA::PSAD-T::::*<br>*ARpro::roGFP2-Tsa2ΔC_R::SV40::RPL23-T*	2	This study

pMBS655	*PSADpro::aadA::PSAD-T:::: ARpro::HSP70C::roGFP2-Tsa2ΔC_R::MStop::RPL23-T*	2	This study
pMBS656	*PSADpro::aadA::PSAD-T:::: ARpro::BIP1::roGFP2-Tsa2ΔC_R::ER-ret::RPL23-T*	2	This study
pMBS473	*PSADpro::aadA::PSAD-T:::: ARpro::roGFP2::MStop::RPL23-T*	2	This study
pMBS720	*PSADpro::aadA::PSAD-T:::: ARpro::roGFP2-Orp1::MStop::RPL23-T*	2	This study

Supplemental Table S3. Transgenic lines generated, number of transformants analyzed, and number of localizations observed in different cells.

Trans-genic line	Transformed plasmid	Localization	Number of transformants analyzed	Number accumulating sensor to readily detectable levels and with the expected size	Number of cells with this localization per number analyzed
ARpro	pMBS472	cytosol	13	6	17/17
PSADpro	pMBS431	cytosol	13	9	7/7
USPA	pMBS650	stroma, cytosol	13	6	19/19
70B	pMBS651	stroma, cytosol	13	3	19/19
CDJ1	pMBS652	stroma	12	9	15/15 (aggregates)<br>10/10 (no aggregates)
PSAN	pMBS653	thylakoid, stroma, cytosol,	13	9	11/11
PSBO	pMBS654	thylakoids	13	8	50/50
SV40	pMBS657	nucleus	13	7	7/7
70C	pMBS655	mitochondria	13	4	28/28
BIP1	pMBS656	ER	13	7	23/23
roGFP2	pMBS473	cytosol	13	9	4/4
Orp1	pMBS720	cytosol	13	6	12/12
