# Supplementary material for: Real-time monitoring of subcellular H2O2 distribution in Chlamydomonas reinhardtii
Source: Plant Cell. 2021 Jul 1;33(9):2935–49. doi: 10.1093/plcell/koab176 (PMC8462822; doi:10.1093/plcell/koab176)
Supplement: koab176_Supplementary_Data [file koab176_supplementary_data.zip › tpc.00945.2020-s03.pdf]

Real-time Monitoring of Subcellular H_2O_2 Distribution in *Chlamydomonas reinhardtii*

Justus Niemeyer, David Scheuring, Julian Oestreicher, Bruce Morgan, and Michael Schroda

Corresponding authors: *Michael Schroda* *schroda@bio.uni-kl.de*
Bruce Morgan *bruce.morgan@uni-saarland.de*

Review timeline:

**TPC2020-RA-00945**	Submission received:	Nov. 13, 2020
	1st Decision:	Dec. 19, 2020 *decline*
**TPC2020-RA-00945-A**	Appeal received:	Jan. 05, 2021
**TPC2020-RA-00945R1-A**	1st revision received:	Feb. 22, 2021
	2nd Decision:	Apr. 01, 2021 *request revisions*
**TPC2020-RA-00945R2-A**	2nd revision received:	May 24, 2021
	3rd Decision:	May 28, 2021 *accept*
	Final acceptance:	Jun. 28, 2021

REPORT: (The report shows the major requests for revision and author responses. Minor comments for revision and miscellaneous correspondence are not included. The original format may not be reflected in this compilation, but the reviewer comments and author responses are not edited, except to correct minor typographical or spelling errors that could be a source of ambiguity.)

**TPC2020-RA-00945**	**Submission received**	**Nov. 13, 2020**

Thank you for choosing to send your manuscript entitled "Real-time monitoring of subcellular H_2O_2 distribution in *Chlamydomonas reinhardtii*" for consideration at The Plant Cell. Your submission has been evaluated by members of the editorial board as well as expert reviewers in your field, and we regret to inform you that we are not able to recommend publication of this manuscript. We have not made this decision lightly. We have had input from multiple scientists, and have solicited post-review comments as well. Our present policy is to offer streamlined decisions and to not advise on the direction of the work by requesting extensive modifications or substantial additional experiments.

All 4 reviewers found the manuscript interesting, but all voiced major concerns and highlighted a number of deficiencies, leading us to conclude there are too many unknowns, and the work is too preliminary for publication in The Plant Cell.

Some of major concerns include: a lack of data on the mechanism of reduction of oxidized roGFP (whether achieved via reduction of TSA or directly by accepting electrons from a suitable donor) and lack of thiol quenching kinetics of the roGFP-TSAdeltaCR ex vivo (Reviewer 1), an open question of whether aquaporins in the chloroplast are required for H_2O_2 diffusion out of the chloroplast to the cytosol, and lack of data on the role of cytosolic H_2O_2 scavenging mechanisms in protecting the nuclei, for example using mutants and/or inhibitors (Reviewer 3), and the omission of a peroxisomal probe (the rationale for not including this organelle in the toolbox is unclear), and lack of data showing that the applied concentrations of MV and metronidazole are not reaching toxic levels, leading to extra/artifactual ROS levels after cell death (Reviewer 4).

In post-review consultation, the reviewers further agreed that the mechanism of adjusting the redox state of the probe (including the reduction pathways) needs to be more fully understood to validate the reliability of the probe. In addition, the compartment-specificity of reduction remains elusive. Solely discussing these issues would be insufficient to allay concerns; if the sensor mechanism is questionable, its applicability will be doubtful as well.

Therefore we cannot recommend resubmission of this work. The reviewers point out a number of areas in which the work could be strengthened, which may be helpful to you as you continue your project or revise your manuscript for submission elsewhere. If you decide to complete the story and resubmit to The Plant Cell, it will be evaluated as a new submission subject to full assessment by the editorial board, including pre-review and editor selection, and if sent for external review, a new set of reviewers is likely to be chosen.

Of course, this decision requires judgment and we recognize that we may sometimes mis-judge important work or misinterpret its scope. We hope that this is not the case here, but we are willing to hear thoughtful rebuttals after you have had some time to digest these comments.

TPC2020-RA-00945R1-A 1st Revision received Feb. 22, 2021

-- Reviewer comments (with **Authors responses in bold**):

Following our appeal to the unfair comments of Reviewers 1 and 3, the Editorial Office of The Plant Cell has allowed us to hand in a revised manuscript based on the constructive critics raised by Reviewers 2 and 4. All answers based on new experimental data are given in blue. Since Reviewers 2 and 4 have seen the comments of Reviewers 1 and 3, we decided to also provide our responses to their comments from the rebuttal letter in this point-by-point response to Reviewer's critics. Please notice that we have added experiments to address some of the critics raised by Reviewer 1 regarding his/her concerns on the efficiency of the NEM trapping agent (new Figure 3, showing how fast NEM penetrates the cells and efficiently traps the sensor's redox state) and of Reviewer 3 regarding his/her statement that our sensors are only an 'improvement of existing probes' (new Supplemental Figure 3 where we show how poorly the existing roGFP2-Orp1 sensor performs in Chlamydomonas).

Comments to Authors (*Reviewer 1*):

The topic of hydrogen peroxide signaling is important and the approach reasonable. The generation of flexible modules for targeting the various subcellular compartments is attractive. The execution of the cloning, transformation and analysis appears well-done.

Point 1. *However, there are some drawbacks associated with the constructed probes, the readouts and the interpretations. The TSA2deltaCR variant lacks the resolving Cys, but still it is rereduced in the cellular context.*

The nature of the re-reduction pathway appears entirely or mostly unknown. The compartments of plant cells are packed with possible and putative reducing entities. These complex networks differ between compartments. The used sensor functions as kinetic sensor being balanced between oxidation by H_2O_2 and reduction by unknown electron donors. The efficiency of the electron donors and the oxidation rate by H_2O_2 will vary with physiological conditions, NADPH/NADP ratios, metabolic activities and thus with temperature or photosynthetic active radiation.

The kinetics, specificity and metabolic and temperature-dependencies of re-reduction in vivo essentially is a black box. They also were not investigated in vitro. Given these uncertainties, the meaning of the readouts remains to a significant part elusive.

The critical assessment is supported by the fact that the sensor does not provide useful information from the ER with likely lacking regeneration system proving that the regeneration systems and their catalytic efficiencies are of ultimate important.

RESPONSE. It is important to point out that roGFP2-Tsa2ΔC_R has been extensively characterised in other organisms, for example in *S. cerevisiae*, where it was first applied, and *C. elegans* (Morgan et al, Nature Chemical Biology, 2016 and De Henau et al, Developmental Cell, 2020). The deletion of the resolving cysteine (C_R) was an intentional step in probe development, as it was shown to strongly limit direct reduction of Tsa2, for example by thioredoxins (Morgan et al, Nature Chemical Biology, 2016). In the cytosol and mitochondrial matrix of yeast cells, roGFP2-Tsa2ΔC_R reduction was shown to be exclusively dependent upon the Grx/GSH mediated reduction of the roGFP2 moiety. We feel that the reviewer has missed the point of the probes and the mechanism by which they are known to function. The reviewer's 'broad-brushed' dismissal of the probes' utility and ability to uncover new biology is extremely disappointing. It completely ignores the large impact that redox sensors have had on our understanding of cellular redox biology in the past decade, reflected in numerous new discoveries and high-impact publications. The dismissal of the roGFP2-Tsa2 probe with the statement that 'the meaning of the readouts remains to a significant part elusive' seems to be based on a misunderstanding of the probe's mechanism and capabilities and is therefore very disappointing.

It is important to reiterate what roGFP2-Tsa2ΔC_R can, and cannot, do. RoGFP2-Tsa2ΔC_R is not, and cannot be, a quantitative H_2O_2 sensor. Neither we, nor anyone else, have claimed that it can quantitatively report cellular H_2O_2 levels. This is not a specific limitation of our sensor but applies generally to all currently available genetically encoded or small chemical H_2O_2 sensors. Indeed, the oxidation of the sensor by H_2O_2 and its reduction by GSH/glutaredoxin (or putatively by some other redox protein) reflects how the oxidation of any H_2O_2-reactive protein thiol in the cell is controlled, i.e. by both H_2O_2-dependent oxidation and GSH/Grx (or Trx for other protein thiols, but not for roGFP)- dependent reduction. Given these considerations it is not possible to compare probe oxidation between different subcellular compartments and to subsequently draw conclusions from this data about the relative absolute H_2O_2 concentrations in each compartment. We do not do this! However, it is possible to monitor qualitative changes in H_2O_2 levels; we do this! We have monitored compartment-specific probe changes in response to several different H_2O_2 producing treatments. We observed that probe responses were always strongest in compartments nearest to the site of H_2O_2 production/influx and became progressively weaker with increasing distance. It is therefore not the case that a probe in a specific compartment is inherently less responsive. It is from the compartment-specific changes in probe response that we conclude that there must be intracellular H_2O_2 gradients, which are generated via efficient H_2O_2 scavenging. This conclusion is also supported by recent in silico studies and experiments in mammalian cells.

It would of course be desirable to know the specific enzymes that mediate probe reduction in compartments such as the mitochondrial matrix or the chloroplast stroma. However, given the large number of candidate enzymes in these compartments, the current limitations of Chlamydomonas genetics, and the possibility for redundancy between reductive systems, to address this question at present would be extremely challenging and would probably require a deeper understanding of Chlamydomonas redoxins than we currently have. In summary, we are confident that the relative responses of the roGFP2-Tsa2ΔC_R probe in different subcellular compartments, in different experimental setups, is completely consistent with the precise localisation of H_2O_2 production or exogenous application and its subsequent diffusion. There is

nothing in our data to suggest that there are significant subcellular compartment-specific differences in the absolute H_2O_2 sensitivity or response kinetics of roGFP2-Tsa2ΔC_R.

Finally, we would like to re-iterate again the advantages of our sensor. There are three major advantages over previous sensors: 1. Ultra-sensitivity; sufficient to report changes in 'basal' H_2O_2 levels in specific subcellular compartments. 2. Reversibility, in contrast to all small chemical H_2O_2 probes/dyes. This allows for fully dynamic monitoring of changes in subcellular compartment specific H_2O_2 levels. 3. Subcellular compartment specificity, in contrast to all small chemical H_2O_2 probes/dyes, our sensor, in common with other genetically encoded probes, can be targeted to any subcellular compartment.

Point 2. *What is the mechanism of reduction of oxidized roGFP? Is it achieved via reduction of TSA or directly by accepting electrons from a suitable donor?*

RESPONSE. As stated above, all the data available indicate that direct reduction of the sulfenic acid on Tsa2 is not an efficient means of probe reduction. Probe reduction has been shown to be dependent upon GSH/Grx in other organisms and this is very likely the dominant mechanism for reduction in the cytosol in Chlamydomonas. We are aware that enzymatically active Grxs have not been reported to be present in the mitochondrial matrix and chloroplast of Chlamydomonas, thus leaving the door open for alternative redox enzymes to be involved in reducing roGFP2-Tsa2ΔC_R. However, even if this is the case, we consider that the identity of these enzymes would be a 'nice to know - although with the limited knowledge of Chlamydomonas redox biology in general and the very limited genetic tools available this would be extremely difficult to achieve' but would not affect the oxidation of the roGFP2-Tsa2ΔC_R probe. This statement is supported by all the previous characterisation work performed on this sensor as well as related redox enzyme-roGFP constructs. It is also evident, by simple comparison of the oxidation and reduction kinetics in each subcellular compartment, that oxidation proceeds several orders of magnitude more rapidly than subsequent reduction, as described above and as reported in several previous studies.

Point 3. *With this said, the heading in l.238 may be correct, likely is correct, but the data do not prove this conclusion. If the re-reduction rates differ between different compartments, then the sensors would theoretically respond with distinct kinetics even at the same H_2O_2-concentration.*

RESPONSE. As we outlined in detail above. Due to the kinetics of oxidation of roGFP2-Tsa2ΔC_R it is extremely unlikely that the reductive reaction will affect either the sensitivity towards H_2O_2 or the oxidation kinetics. We therefore are confident that similar concentrations of H_2O_2 are required to elicit a roGFP2-Tsa2ΔC_R response in each subcellular compartment. Therefore, comparison of probe responses does permit qualitative statements to be made concerning the relative amount of H_2O_2 reaching each compartment.

Point 4. *line.57: the statement that H_2O_2 is stable is correct in comparison with other reactive oxygen species. But the entire cell is filled with all kind of non-enzymatic or enzymatic antioxidants which counteract accumulation of hydrogen peroxide. The statement on the suitability of H_2O_2 as second messenger is not as black and white as written in this section.*

RESPONSE. This is another perplexing and 'broad brushed' statement apparently questioning the role of H_2O_2 as a second messenger. The role of H_2O_2 in cellular redox signalling is supported by

hundreds of publications from many research groups. Clearly, many mechanistic details remain unknown and are often highly nuanced, for example many supposed H_2O_2 scavenging enzymes may be crucial transducers and transmitters of H_2O_2 signals. However, we did not feel that the introduction of a technical manuscript on a redox sensor was the place to get into detailed discussion on the mechanism of H_2O_2 signaling.

Point 5. *l.256: The NEM trapping is interesting. But how can its reliability be proven? How rapid does NEM (40 mM) diffuse into the cell. Thiols are not equally reactive with NEM. It may be the good news, that the TSA thiolate may be particularly sensitive to NEM quenching? It would have been more convincing to see some thiol quenching kinetics of the roGFP-TSAdeltaCR ex vivo.*

RESPONSE. The Tsa thiolate would not be relevant here, the roGFP2 cysteine needs to be alkylated to trap the probe redox state and block further fluorescence changes. Nevertheless, we have conducted an experiment that impressively shows how fast the addition of NEM leads to the "freezing" of the redox state of roGFP2 (new Figure 3).

Comments to Authors (*Reviewer 3*):

Point 1. *This is a very interesting paper that describes the dynamics of H_2O_2 metabolism and signaling in Chlamydomonas in response to high light and heat stresses. Because I am sure this paper is going to get scrutinized to the extreme by the roGFP Tsars, that are desperately trying to shoot down any paper that competes with them and their work, I will focus in my review on the significance of the scientific discoveries the authors made (and leave any technical issues that may arise to be handled by some of the other reviewers).*

RESPONSE. We submitted this manuscript as a technical advance reporting on the development of methodologies to permit the use of an ultra-sensitive genetically encoded H_2O_2 sensor in Chlamydomonas, we are perplexed that the reviewer can ignore the technical aspects of the work, when this is the main focus of the paper.

Furthermore, the reviewer's comment '*scrutinized to the extreme by the roGFP Tsars, that are desperately trying to shoot down any paper that competes*' implies that the reviewer is very bitter towards the roGFP-sensing field, yet also reveals that he/she does not even know who the main players in this field are. The second corresponding author on our manuscript, Bruce Morgan, has been involved in the generation of more roGFP2-based probes than anyone else, see for example,

Morgan et al, Nature Chemical Biology, 2013

Morgan et al, Nature Chemical Biology, 2016

Staudacher et al, Redox Biology, 2017

Roma et al, Antioxid. Redox Signal., 2018

Calabrese et al, EMBO Journal, 2019

Liedgens et al, Nature Comms, 2020

Zimmermann et al, Redox Biol, 2020.

Amponsah et al, Nature Chemical Biology, 2021.

Bruce Morgan has published extensively with anyone that might be considered a 'roGFP Tsar' by the reviewer, for example, Tobias Dick, Andreas Meyer, Markus Schwarzländer, all of whom are extremely valued mentors and collaborators. Rather than 'desperately trying to shoot down' our

manuscript, we are confident that anyone from the roGFP-field would be happy to provide a letter of support for our manuscript upon request.

Point 2. *Ever since the discovery of H_2O_2 production in the chloroplast during photosynthesis, it was assumed that H_2O_2 that is made in the chloroplast during the Mehler reaction diffuses out of the chloroplast and impacts signaling and stress at the cytosol and nuclei. The recent work of Exposito-Rodriguez et al., 2017 supported this.*

It is my opinion however that "diffusion" is not the right term to use here since H_2O_2 is most likely transported through aquaporins across membranes...

In their work, the authors have confirmed the chloroplast to cytosol H_2O_2 transport again. In contrast, however, the authors now show that H_2O_2 produced in the chloroplast during photosynthesis does not reach the nuclei and is hypothetically scavenged in the cytosol. In contrast to the light stress experiment, the authors report that heat stress results in H_2O_2 accumulation in the cytosol and not the chloroplast.

The finding that H_2O_2 diffuses out of the chloroplast to the cytosol is not new. The finding that it is scavenged in the cytosol (and this scavenging can be overcome by accelerating the production of H_2O_2 in the chloroplast by for example paraquat) and does not make it to the nuclei is new and contradicts the work of Exposito-Rodriguez et al., 2017.

RESPONSE. This is not correct. Exposito-Rodriguez have shown that the transmission of H_2O_2 from chloroplasts to the nucleus requires a sub-population of chloroplasts closely associated with nuclei, avoiding the cytosol. As we have stated, this is not possible given the architecture of a Chlamydomonas cell with a single, large, and immotile chloroplast.

Point 3. *I am not sure if the manuscript provides a large enough advancement in our understanding of H_2O_2 metabolism and signaling to merit publication in TPC. The probes used are an improvement of existing probes but the results regarding H_2O_2 in the nuclei during light stress needs more work.*

For example, the authors should consider testing whether aquaporins in the chloroplast are required for H_2O_2 diffusion out of the chloroplast to the cytosol. In addition, they could study the role of cytosolic H_2O_2 scavenging mechanisms in protecting the nuclei. These aquaporin/scavenging studies could be done using mutants and/or inhibitors and would enhance our understanding of H_2O_2 dynamics in plant cells.

RESPONSE. As pointed out above, this work was submitted as a breakthrough report on a sensor that is ~20-fold more sensitive than those used so far in plants and the first H_2O_2 sensor described for the widely used model system Chlamydomonas. In new Supplemental Figure 3 we show that the 'existing sensor' roGFP2-Orp1 expressed to similar levels as roGFP2-Tsa2ΔC_R in the Chlamydomonas cytosol barely shows a response to exogenously added H_2O_2, therefore confirming the extraordinary sensitivity and superior performance of roGFP2-Tsa2ΔC_R. Hence, the establishment of roGFP2-Tsa2ΔC_R is definitely much more than an 'improvement of existing probes'. The applications of the new sensors are proofs of principle showing their high potential for new discoveries in plant biology. Understanding H_2O_2 metabolism and signaling or the roles of aquaporins in H_2O_2 diffusion is far beyond the scope of the manuscript.

Point-by-point responses to Reviewers 2 and 4:

We would like to thank both reviewers for their constructive critiques. We have fully addressed them with additional data/experiments as outlined below.

Comments to Authors (*Reviewer 2*):

Point 1. *The paper entitled « Real-time monitoring of subcellular H_2O_2 distribution in* Chlamydomonas Reinhardtii *» by Niemeyer and colleagues describes the expression of a genetically encoded, fluorescent H_2O_2 sensor (roGFP2-Tsa2ΔCR) in major subcellular compartments of Chlamydomonas cells. The study demonstrates that these sensors respond to both exogenously added and endogenously produced H_2O_2 (in the case of high light or heat stress treatments) thus allowing real-time monitoring of H_2O_2 production.*

This study is of high novelty and describes a promising tool for assessing intracellular H_2O_2 homeostasis and dynamics in various conditions in this cellular model. The paper is extremely clearly written and I have no real concern about the experiments.

In Fig 1D, I wonder why there is no fluorescence image for the PsaD*-driven expression of the cytosolic roGFP2-Tsa2ΔCR -3X HA construct, as done for all other tested constructs. Also, in this figure, I am puzzled by the punctate signal obtained for the* CDJ1*-driven chloroplastic roGFP2-Tsa2ΔCR construct. Is there any reason for that?*

> **RESPONSE. As requested, we have included an image of the *PSAD* promoter-driven sensor in revised Figure 1D. The punctate signal observed for the stromal sensor targeted by the CDJ1 signal peptide is well observed and surprised us, too. It might be derived from the formation of heterooligomers of roGFP2-Tsa2ΔC_R with stromal peroxiredoxins, as has been reported by Morgan et al., 2016, Nat Chem Biol. We have mentioned this possible interpretation in the revised text and analysed a transformant expressing the stromal sensor at weaker levels. In this line, we do not see the punctate signals in the stroma, but a similar (albeit noisier) response to exogenously added H_2O_2 as for the line with higher expression levels (new Supplemental Figure S2).**

Point 2. *In fig. 2, do the authors want to speculate about a link between the lower reduction/recovery rate of the mitochondrially-expressed roGFP2-Tsa2ΔCR sensor and the absence of redox active glutaredoxin in mitochondria ? This may also applies to the thylakoid lumen targeted sensor.*

> **RESPONSE. Please see our comments to Reviewer 1 above. Yes, we do consider it a possibility that the lack of a redox active glutaredoxin in these compartments might affect the reduction rate. However, it is important to point out that all the data we have from this study and from previous studies of both this probe and other roGFP2 fusions, indicate that changes in the reductive reaction would not affect the very rapid kinetics of probe oxidation. In other words, changes in roGFP2 reduction, which occurs over a timescale of tens of minutes, would not affect the sensitivity of the probe response to rapid H_2O_2-dependent oxidation, occurring on a seconds to minutes timescale.**

Point 3. *Comparing figures 2 and 3, it appears that the initial oxidation state of the sensors at the zero time point is much higher in the high light treatment (figure 3) as compared to the exogenous H_2O_2 application (figure 2). It would be useful to state somewhere why this is the case. Have the cells been cultivated under the same low light regime before starting both experiments? Perhaps an effect of the cell centrifugation ?*

RESPONSE. We also observed this phenomenon in yeast (Morgan et al, Nature Chemical Biology, 2016). Upon concentration of the cells in the wells of a microtiter plate, we observe a rapid, cell-dependent, consumption of oxygen, a concomitant decrease in H_2O_2 levels and thus also a decrease in probe oxidation. The preparation time of the microtiter plates is in the range of 15 minutes, which gives plenty of time for a decrease in apparent probe steady state oxidation. Thus, the NEM-trapping reveals the true probe steady state oxidation, the starting point of the dynamic assays does not. We have added two sentences in the revised manuscript to point this out. Confirmation of this effect in yeast came from experiments using respiratory deficient yeast, where the probe steady state oxidation is the same in both NEM trapping and plate-reader experiments.

Minor points :

Point 4. *Line 135. Define RBCS2 for non-specialists*

RESPONSE. Done.

Point 5. *Line 595-597 : I wonder whether the equation is correct. I suspect that in the last part of the denominator either something is missing or there is an extra - symbol.*

RESPONSE. Very well observed, thank you! There were indeed several errors in the equation. Nonetheless, the correct equation was used for all calculations in our work.

Comments to Authors (*Reviewer 4*)

In Niemeyer et al., the authors take us on their journey of the development of an interesting toolkit to monitor H_2O_2 levels in different subcellular compartments of Chlamydomonas. They carefully describe the development and testing, including those that failed or performed suboptimal, of various roGFP-TSA2 based constructs. They nicely show that exogenous H_2O_2, HL stress, MV and heat stress are affecting -to a different degree- H_2O_2 levels in the different compartments. Interesting is the discrepant observation (compared to the previously published observations in Arabidopsis) that nuclear H_2O_2 levels are not altering upon HL stress. This is an interesting report on toolbox which is prone for further detailed explorations.

Remarks

Point 1. *For the non Chlamy expert, the description of the cloning effort might be too technical. E.g. level 0, level 1 warrant better explanation.*

RESPONSE. This is true. We have included two sentences at the beginning of the Results section to briefly explain the MoClo system. Note, however, that this system is not unique to Chlamydomonas. Because of its amazing power it is rapidly expanding to all kind of model organisms.

Point 2. *In the Reference list Cheeseman et al seems incomplete*

RESPONSE. Indeed, page numbers were missing, this has been fixed.

Point 3. *Unfortunately a peroxisomal probe is missing. What is the rationale not including this organelle in the toolbox?*

The Chlamydomonas MoClo kit (Crozet et al., 2018) indeed contains a part that allows targeting of proteins to the peroxisome-like microbodies (this is the PTS1-like sequence from the C-terminus of Chlamydomonas malate synthase, -HIVTKTPSRM-). This sequence, added to GFP or mVenus, allowed their targeting to microbodies (Hayashi and Shinozaki, 2012, J Plant Res; Crozet et al., 2018). We therefore used this MoClo part and added it to the C-terminus of roGFP2-Tsa2ΔC$_{R}$. As shown below, we indeed obtained several transformants that expressed the protein to high levels. However, CFLS microscopy revealed a cytosolic localization of the sensor in four transformants. Sequencing of the Level 2 construct used for transformation revealed the correct sequence. Hence, the PTS1-like sequence does not mediate the targeting of our sensor to microbodies. Why this is the case is not clear. Perhaps it is related to the larger size of the sensor? However, since this is a negative result, we chose not to include these data in the manuscript.

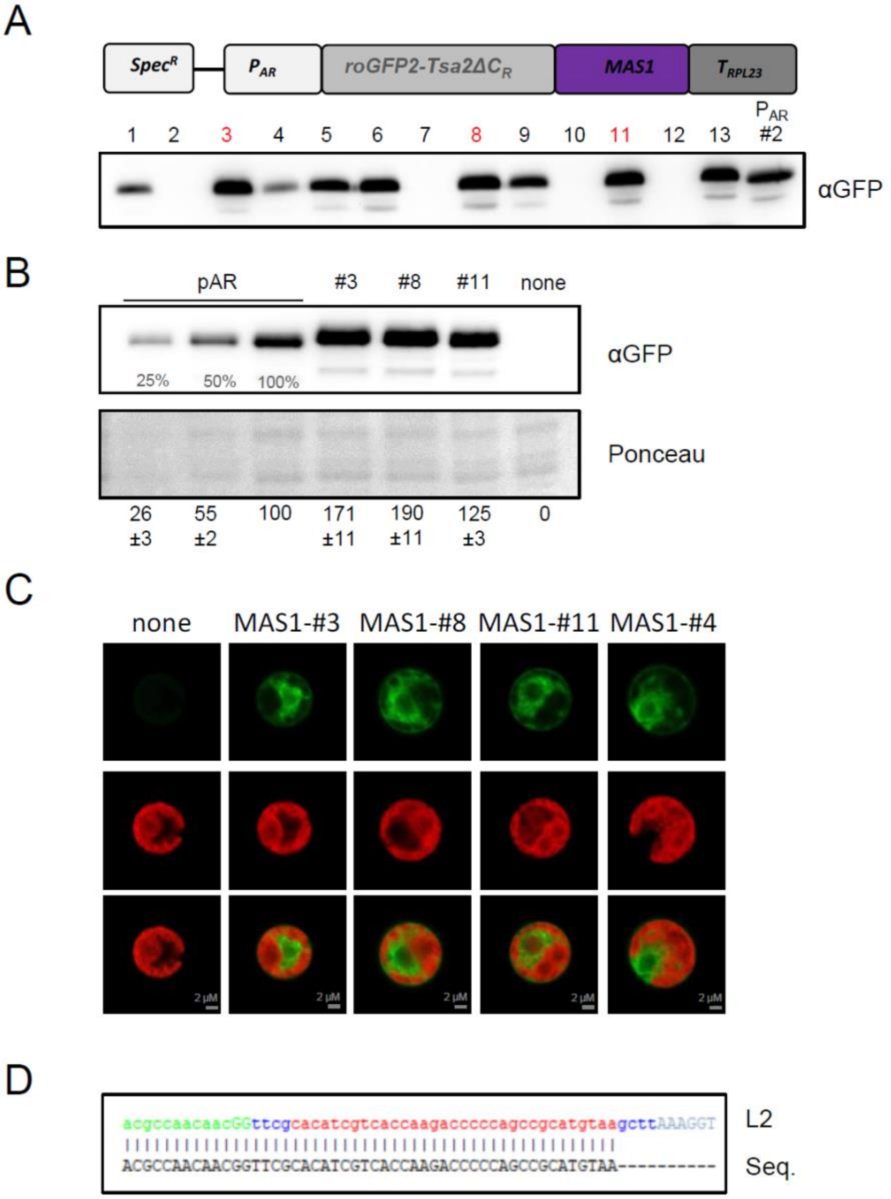

Point 4. *For the heat stress experiments, only the cytosolic version was tested. Would be of interest to see how the other compartments behave.*

> **RESPONSE. A very good point. We did the experiment with NEM trapping also for sensors in stroma, matrix, and nucleus (new Figure 5B). Strikingly, a significant increase in H_2O_2 levels upon heat stress was also detected in the nucleus and a trend to increased levels was observed in mitochondria, while H_2O_2 levels in the stroma declined. These results confirm the DCMU experiments indicating that heat stress induced H_2O_2 does not derive from the chloroplast. A likely source is the NADH oxidase in the plasma membrane, but other sources are possible.**

Point 5. *It would be useful to check whether the applied concentrations of MV and metronidazole are not reaching toxic levels, leading to extra/artifactual ROS levels after cell death.*

> **RESPONSE. This is a valid point. We tested the toxicity of both drugs via growth assays. For this, we split two cultures in three subcultures of which one each was left untreated, and two each were supplemented with 2 mM MV or 1 µM metronidazole for 1 h. Then all cultures were centrifuged, cells washed and resuspended in fresh medium. To one of the cultures treated before with a drug, the respective drug was re-added. Then we monitored growth over time. This was done with three independent replicates. As shown in new Supplemental Figure 4, we observed a growth arrest in cultures continuously exposed to either of the drug. The cultures exposed to the drug for 1 h had a growth retardation but reached similar cell densities in stationary phase as the untreated control. In the experiments shown in Figure 4, we monitored H_2O_2 levels after MV/metronidazole treatment for only 30 min. Hence, we conclude that the viability of the cells was not yet affected by the drugs.**

TPC2020-RA-00945R1-A 2nd Editorial Decision – *request revisions* **Apr. 01, 2021**

We have received reviews of your manuscript entitled "Real-time monitoring of subcellular H_2O_2 distribution in *Chlamydomonas reinhardtii*" (two previous reviewers #2 and 4 and a new reviewer #5) and also engaged in post-review consultation with all three reviewers. Thank you for submitting your best work to The Plant Cell. The editorial board agrees that the work you describe is substantive, falls within the scope of the journal, and may become acceptable for publication, pending revision and potential re-review.

We ask you to pay attention to the following points in preparing your revision:

As stated in the appeal decision letter, we have in particular solicited input from a new reviewer with expertise with sensors (Reviewer #5). As you can see, this reviewer agreed that some of the original reviewer comments were unjustified, and believes the manuscript has considerable value. Nevertheless, this reviewer suggested a round of revision including additional experimental work to make the work suitable for publication in The Plant Cell. These include:

1) repeating experiments present in Figure 5 with the Chlamydomonas strain expressing the Orp1-sensor roGFP2-Orp1,
2) testing the hypothesis for an involvement of NAD(P)H oxidases as suggested, and
3) with respect to additional specific comments on Figures 2 and 4, independent confirmation of the steady state OxD level for Tsa2ΔC_R in Chlamydomonas as suggested.

This reviewer believed the work requested to be "feasible and straightforward." For example, it is shown in the supplement that roGFP2-Orp1 is insensitive to H_2O_2 in this experimental system. This feature might be exploited to

distinguish between oxidation of Tsa2ΔC$_R$-roGFP2 by H_2O_2 and oxidation by GSSG. The latter should act on both probes in a similar way while the sensitivity appears to be different. So, if roGFP2-Orp1 in contrast to Tsa2 does not respond to heat a logical conclusion would be that the oxidation of Tsa2 indeed depends on H_2O_2.

With respect to the involvement of NAD(P)H oxidases, multiple possible sources for H_2O_2 production under heat stress are mentioned in the manuscript. The photosynthetic electron transport chain is not necessarily the critical source, especially not in cells that are not illuminated beyond the requirement for the roGFP excitation. The pharmacological experiments proposed would address this question. If, for example, inhibition of NADPH oxidases by DPI diminishes heat-induced H_2O_2 production, this would strengthen the argument, and conversely, if it does not have an effect, the results would be starting points for (future) follow up work.

Please consider all remaining comments of Reviewer 5 as well. After studying the review comments, we hope that you will be able to address some or all of the requests of reviewer 5. However, we are willing to consider thoughtful author responses to the question of the feasibility and value gained in adding the requested experimental work.

TPC2020-RA-00945R2-A 2nd Revision received May 24, 2021

-- Reviewer comments (with **Authors responses in blue**):

We are grateful to this Reviewer for the critical and constructive critiques. They have been extremely valuable to further improve our manuscript.

Reviewer #5 (Comments for the Author):

The dynamics of H_2O_2 in cells exposed to abiotic or biotic stress and the role of H_2O_2 as a putative messenger has been discussed for decades. Improved molecular tools for dynamic monitoring of H2O2 have been developed and continuously improved over the past 15 years. Here a peroxiredoxin-based probe, Tsa2ΔC$_R$-roGFP2 has been expressed and successfully used in a photosynthetically active organism. The work confirms the original characterization of this probe variant being more sensitive than its predecessors. A ~20-fold improvement in sensitivity is certainly a quantum leap that deserves attention as it may indeed offer opportunities for new insights into plant redox signaling in the future.

The manuscript has undergone a first round of reviewing and subsequent revision. The authors appealed against two reviews (out of 4) because of some rather harsh and obviously rather subjective if not incorrect comments by the respective reviewers. Nevertheless, not all those comments were unjustified and indeed the authors considered some points in their revision to further improve the manuscript.

Point 1. One major issue (also raised by reviewer #1 in the first round) is the uncertainty about the readout regarding oxidation of the probe by H_2O_2 and reduction via glutathione. This has implications for the readout and its interpretation. It is widely accepted this probe, like any other genetically encoded H_2O_2 probe, cannot be used for quantitative measurements of H_2O_2 in any subcellular compartment. This is correctly not claimed in the manuscript. In the experiments testing the probe's sensitivity to externally applied H_2O_2 (Fig. 2) it is highly likely that the probe primarily responds to H_2O_2. The same is

true for light-induced oxidation, which can be prevented at least partially through inhibition of the photosynthetic electron transport chain. For the heat stress experiment, however, this is less clear. The oxidation of Tsa2ΔC$_R$-roGFP2 in response to a temperature increase from 23ºC to 40ºC may well be caused by increased levels of H_2O_2, but a partial oxidation of the glutathione pool and hence diminished reducing power for the continuous reduction of roGFP2 cannot be fully excluded. Given that the authors do have a Chlamydomonas strain with expression of roGFP2-Orp1, the experiments presented in Fig. 5 should be paralleled by similar measurements with the Orp1-sensor. Given that Orp1-roGFP2 is far less sensitive to H_2O_2 (as shown in the supplemental Fig. 3) but should have the same sensitivity to the glutathione redox potential such an experiment should be suitable to minimize the ambiguities.

RESPONSE: The proposed experiment is an excellent idea. As suggested, we have performed it with the roGFP2-Orp1 sensor targeted to the cytosol using NEM-trapping. As shown in new Supplemental Figure 8, the oxidation state of the roGFP2-Orp1 sensor did not change under heat stress. This supports the interpretation that increased oxidation of the roGFP-Tsa2ΔCR sensor under heat stress is mediated by increased levels of H_2O_2 and not by a possibly diminished power of the reducing systems driving roGFP2 reduction.

Point 2. As the authors emphasized in their rebuttal, this submission is meant to be a technical paper. While this is absolutely correct some obvious biological questions arising from the use of the probe should be tackled if it can be done easily. Indeed, it was tested whether the oxidation observed in the cytosol during heat stress is causally connected to activity of the photosynthetic electron transport chain. With that piece of data shown it is not plausible why the second biggest hypothesis for an involvement of NAD(P)H oxidases was not tested in a similar way. RBOH-like proteins in Chlamydomonas very likely would be inhibited by diphenyleneiodonium (DPI) and thus similar experiments like those done with DCMU ought to be straightforward. The frequent argument that DPI is not sufficiently specific and would also inhibit other Flavin-dependent enzymes including glutathione reductase does not hold true if parallel measurements with roGFP2-Orp1 are done as outlined above.

RESPONSE. This experiment is another excellent idea. We have performed it with the cytosolic roGFP-Tsa2ΔCR and roGFP2-Orp1 sensors using NEM-trapping. As shown in new Supplemental Figure 8, we observed an increased oxidation of both sensors after the addition of DPI at a concentration of 10 µM under nonstress and heat stress conditions. This indicates that DPI impairs the systems driving roGFP2 reduction and perhaps also leads to an increased production of H_2O_2. To rule out that this effect was caused by too high concentrations of DPI, we monitored the oxidation state of the more sensitive roGFP-Tsa2ΔCR sensor at lower DPI concentrations (also in new Supplemental Figure S8). We found no effect at DPI concentrations of 2.5 µM and below, while sensor oxidation increased significantly at concentrations > 5 µM. Hence, DPI has no effect on heat stress induced H_2O_2 production in Chlamydomonas and apparently is no suitable drug to study a potential role of NAD(P)H oxidases in H_2O_2 production under heat in combination with our sensors in this organism.

Specific concerns:

Point 3. Line 49: 'algal and plant cells': So far, no one has reported successful expression of the Tsa2 probe in higher plants. 5 years after the original report this is a bit surprising because typically many people jump on such new tools. Given that fact that the probe cannot easily be expressed and used in mammalian cells and in the light of potential problem resulting from oligomerization with endogenous

peroxiredoxins, I feel that it would be appropriate not to claim the potential impact for research on higher plants prematurely.

> **RESPONSE. Indeed, we heard from colleagues working on Arabidopsis that they had trouble getting the sensor expressed. Since it is not clear whether these problems can be solved, we changed the sentence to refer to plant cells in general, including cells from alga and higher plants.**

Point 4. Line 160: The HA tag was not mentioned before and thus is a bit surprising here. It should be mentioned further up that the PSAD constructs carries a 3xHA, which then also more easily explains the mass shift seen in Fig. 1C. At the moment the mass shift is not explicitly explained.

> **RESPONSE. We have introduced the use of the 3xHA tag when describing the different parts and mention that it causes the size shift in the *PSAD* promoter driven construct.**

Point 5. Line 178-181: The punctate structures - likely protein aggregates - are unwanted artefacts, which may indeed occur more frequently at higher expression levels. The supplement shows the same construct with lower expression levels and without any obvious aggregates. With such an obvious artefact I wonder why the artefact is shown in the main text and the strain without hidden in the supplement.

Would it not make sense to present the data just the other way round?

> **RESPONSE. The reason is that the fluorescence signal in the line expressing the stromal sensor to higher levels is much less noisy. Since the quality of the response to exogenous H_2O_2 was the same, we gave higher priority to the cleaner signal in the line forming the aggregates. We looked at expression levels of cytosolic chaperone HSP70A, stromal protease DEG1C, chaperone HSP22E/F, disaggregase CLPB3, and membrane stress marker VIPP2 and found no change in their levels in the aggregate-forming transformant. Hence, these aggregates appear not to trigger an unfolded protein response and we consider it safe to use this transformant line.**

Point 6. Line 208; Fig. 2A: The data for OxD apparently become negative and were cut off. This of course doesn't make sense. Ideally, raw data for individual channels should be shown in the supplement to better evaluate the quality of the data and more specifically identify the relevant bottleneck, which is likely on the 405 nm channel.

> **RESPONSE. This is a valid critique. We repeated the experiment with cells expressing the sensor with the *PSAD* promoter. The signal is still very noisy but there are no negative values anymore. As suggested, we added the recordings of the 405 nm and 488 nm excitation over time in new Supplemental Figure 3. As the reviewer suspected the noise in the OxD data derives mainly from the 405 nm channel.**

Point 7. Line 210: 'more than two-fold': In line 161 it was said "~2-fold higher". Check for consistency.

> **RESPONSE. Very well observed, fixed.**

Point 8. Line 216: Mention that this was the same concentrations as used before.

RESPONSE. Done.

Point 9. Fig. 2 and Fig. 4: The data suggest that at steady state Tsa2 is less oxidized in the nucleus than in the cytosol. How can that be explained?

RESPONSE. This observation is highly reproducible, but we can only speculate on the underlying reasons. Assuming that the main sources of cellular H_2O_2 under steady-state conditions are mitochondria and chloroplasts, H_2O_2 produced there needs to cross the cytosol in order to reach the nucleus. We observed in the HL response that H_2O_2 produced by the chloroplast was detected in the cytosol, but not in the nucleus, presumably because most H_2O_2 is quenched in the cytosol. As a result, we observe the steep H_2O_2 gradients with levels highest in mitochondria and chloroplasts, intermediate in the cytosol, and lowest in the nucleus. Alternatively, there might be even more efficient H_2O_2 quenching systems in the nucleus, potentially combined with more efficient roGFP2 reducing systems.

Point 10. In the plastid stroma, values for OxD after imposed oxidation are significantly below steady state values. Why is this the case?

RESPONSE. The stromal sensor is to ~40% oxidized when we start the recording in the plate reader. Under these conditions, we have a balance between sensor reduction (presumably by glutaredoxins and GSH) and its oxidation via H_2O_2 and Tsa2. The source of H_2O_2 in the light is most likely the Mehler reactions, but there are also reports on H_2O_2 production directly at PSII. Addition of exogenous H_2O_2 leads to further sensor oxidation. Since we monitor cells in the dark in the plate reader, no more H_2O_2 is produced by the Mehler reactions or at PSII, O2 is consumed by respiration and this leads to a depletion of cellular H_2O_2. Since the sensor is continuously reduced and H_2O_2 levels decline in the dark, the sensor eventually becomes more reduced than under steady-state conditions.

Point 11. It is claimed that the initial values in Fig. 4 show the best approximation of the steady state OxD level for Tsa2ΔC_R, while data in Fig. 2 show a lower OxD due to diminished abundance of H_2O_2 resulting from O2 depletion (l. 283-289). This argument is simply derived from earlier observations on yeast. However, I am not fully convinced that this applies to photosynthetically active cells in the same way. What one would like to see is some independent confirmation of this for Chlamydomonas. If this hypothesis is correct, light-induced oxidation of the probe in cells prepared as for the experiments shown Fig. 2, should show a far more pronounced oxidation after illumination than those used in the experiments shown in Fig. 4. Without continuous illumination in the plate reader this might be difficult, but it should be no problem to show this with the NEM blocking approach.

RESPONSE. Since illumination in the plate reader is indeed not possible, we chose to use a similar approach as has been used in yeast, i.e., to demonstrate that the reduction in sensor oxidation over time depends on the number of cells employed. As shown in new Supplemental Figure 4, we observe a faster reduction of sensor oxidation with increasing cell numbers. We demonstrate this for the cytosolic and the stromal sensors.

Point 12. Line 271-277: This is very puzzling in parts: 0.1 mM H_2O_2 were added to the cells. According to Fig. 2B this should lead to a peak OxD of about 0.6 and subsequently a gradual decline. The peak oxidation is much lower here and a decline is barely visible in the green curve.

RESPONSE. The differences in the OxD peak are most likely due to the time lapse of >1 year between the two experiments, during which technical and biological parameters can have drifted. We have therefore repeated the experiments of Figure 2B and Figure 3 (the latter now with 0.5 mM H_2O_2 added) and get very similar results for OxD peaks. The data is much more consistent now.

Point 13. Line 291-293: This statement is an exaggeration. Having seen the difference in cytosolic OxD between Fig. 2B and Fig. 3 and the noise level I don't think that the single value after 20 min, which has apparently not been statistically tested against the preceding values, can be used as an indicator for recovery.

RESPONSE. True. A statistical test against preceding values indeed revealed that there was no significant recovery. We changed the sentence to "with a non-significant trend for probe recovery after 20 min".

Point 14. Line 293; Fig. 4: The data don't support this statement. It thus should be deleted or statistically supported by further measurements. More data along the time course may also help to support the speculative statement. The standard deviation depicted in these figures should at least to some extent correlate with the noise level seen in continuous recordings shown in Fig. 2. Why is the SD shown here for mitochondria (Fig. 4C) so much lower than in Fig. 2F even though this is nominally the same kind of experiment with three independent measurements? This applies to some extent also to the other compartments.

RESPONSE. In the NEM trapping experiments, the value for each time point is the mean of three biological replicates, each of which has been measured 15 times (we have included this information in the Methods section). In contrast, in the real-time measurements each time point consists of three biological replicates that have been measured only once. This explains why in the NEM measurements the variability is purely of biologic origin, while in the real-time measurements it is a combination of biological and technical variability. It is therefore unlikely that we get the values for the mitochondrial sensor to become significant with more replicates and prefer to stick to "the nonsignificant trend".

Point 15. Line 295; Fig. 4B,D,E; Fig. 2: High light results in oxidation of the cytosolic probe but not the nuclear probe (Fig. 4B, D). After addition of metronidazole the oxidation in the nucleoplasm with respect to control values is in relative terms far more pronounced than in the cytosol. On the other hand, it was shown in Fig. 2 that the response of the nuclear probe is much less pronounced when cells are treated with H_2O_2 from the outside. This appears inconsistent and requires further explanation.

RESPONSE. In relative terms yes. But in absolute terms the cytosolic sensor is more oxidized under both treatments, exogenously added H_2O_2 and H_2O_2 produced in the presence of metronidazole in the light. In the latter treatment the path that H_2O_2 must pass in the cell is shorter (from the chloroplast through the cytosol to the nucleus) compared to cell exterior, cytosol, chloroplast, cytosol, nucleus. The longer the passage, the more H_2O_2 can be quenched.

Point 16. Fig. 4E: Paraquat is much better studied as a ROS generator in plastids than metronidazole with a wealth of fully consistent reports. It is hard to understand why paraquat wasn't used as the first

choice for testing the effect of photosynthesis-generated H_2O_2 in all compartments. This selective choice needs to be argued convincingly or data for both inhibitors should be shown.

RESPONSE. The action of both drugs on Chlamydomonas has studied in detail by Schmidt, Matlin and Chua, 1977, PNAS. The find that "even low concentrations of methylviologen kill wild-type cells incubated in darkness, indicating that there are lethal effects of this compound that are not a consequence of its interaction with photosynthetic electron transport". This is the reason why we focused on metronidazole. We have added a sentence in the revised manuscript to point this out.

Point 17. Line 760: metronidazole

RESPONSE. Fixed

Point 18. Line 355: Formally Fig. S5 does not show fluorescence intensities but rather ratio values.

RESPONSE. Fixed.

Point 19. Line 361: Do you mean Figure 4?

RESPONSE. Indeed, fixed.

Point 20. Line 365: Do you mean Fig. 5B?

RESPONSE. Indeed, fixed.

Point 21. Line 427: Everything indicates that $Tsa2\Delta C_R$ is more sensitive to H_2O_2 than roGFP2-Orp1, but it is not sound to compare single cells and whole seedlings in this way as an argument for the higher sensitivity of Tsa2.

RESPONSE. We have pointed out that this comparison might not be entirely fair.

Point 22. Line 641: The equation for OxD given here is different from the equation described in most other papers using roGFP probes. To avoid confusion, it would be worth explaining this briefly or provide at least an appropriate reference describing for this equation.

RESPONSE. In the very first version of the manuscript we had a mistake in the equation. This was noticed by Reviewer #2 and we had the equation already corrected. Nevertheless, we added a reference referring to the review by Meyer and Dick (2010), where the equation is explained in detail.

TPC2020-RA-00945R1-A 3rd Editorial Decision – *accept* **May 28, 2021**

We are pleased to inform you that your paper entitled "Cross-Talk between Clathrin-Dependent Post-Golgi Trafficking and Clathrin-Mediated Endocytosis in Arabidopsis Root Cells" has been accepted for publication in The Plant Cell, pending a final minor editorial review by journal staff. At this stage, your manuscript will be evaluated by a

Science Editor with respect to its presentation of scientific content, compliance with journal policies, and presentation for a broad readership.

Final acceptance from Science Editor **Jun. 28, 2021**
